# Toxicity, recovery, and resilience in a 3D dopaminergic neuronal in vitro model exposed to rotenone

**DOI:** 10.1007/s00204-018-2250-8

**Published:** 2018-06-28

**Authors:** Georgina Harris, Melanie Eschment, Sebastian Perez Orozco, J. Michael McCaffery, Richard Maclennan, Daniel Severin, Marcel Leist, Andre Kleensang, David Pamies, Alexandra Maertens, Helena T. Hogberg, Dana Freeman, Alfredo Kirkwood, Thomas Hartung, Lena Smirnova

**Affiliations:** 1 Center for Alternatives to Animal Testing (CAAT), Johns Hopkins Bloomberg School of Public Health, Baltimore, MD USA; 2 Center for Alternatives to Animal Testing (CAAT) Europe, Department of Biology, University of Konstanz, Konstanz, Germany; 3 The Integrated Imaging Center, Department of Biology, Engineering in Oncology Center and The Institute for NanoBioTechnology, Johns Hopkins University, Baltimore, MD USA; 4 Cyprotex Discovery Ltd., Macclesfield, UK; 5 The Mind/Brain Institute, Johns Hopkins University, Baltimore, MD USA; 6 Department of Environmental Health and Engineering, Johns Hopkins University, Baltimore, MD USA; 7 Department of Neuroscience, Johns Hopkins University, Baltimore, MD USA

**Keywords:** Recovery, Resilience, Cellular defence, Gene response, 3D LUHMES, Neurodegeneration, Rotenone, Pesticide

## Abstract

To date, most in vitro toxicity testing has focused on acute effects of compounds at high concentrations. This testing strategy does not reflect real-life exposures, which might contribute to long-term disease outcome. We used a 3D-human dopaminergic in vitro LUHMES cell line model to determine whether effects of short-term rotenone exposure (100 nM, 24 h) are permanent or reversible. A decrease in complex I activity, ATP, mitochondrial diameter, and neurite outgrowth were observed acutely. After compound removal, complex I activity was still inhibited; however, ATP levels were increased, cells were electrically active and aggregates restored neurite outgrowth integrity and mitochondrial morphology. We identified significant transcriptomic changes after 24 h which were not present 7 days after wash-out. Our results suggest that testing short-term exposures in vitro may capture many acute effects which cells can overcome, missing adaptive processes, and long-term mechanisms. In addition, to study cellular resilience, cells were re-exposed to rotenone after wash-out and recovery period. Pre-exposed cells maintained higher metabolic activity than controls and presented a different expression pattern in genes previously shown to be altered by rotenone. *NEF2L2, ATF4*, and *EAAC1* were downregulated upon single hit on day 14, but unchanged in pre-exposed aggregates. *DAT* and *CASP3* were only altered after re-exposure to rotenone, while *TYMS* and *MLF1IP* were downregulated in both single-exposed and pre-exposed aggregates. In summary, our study shows that a human cell-based 3D model can be used to assess cellular adaptation, resilience, and long-term mechanisms relevant to neurodegenerative research.

**Electronic supplementary material:**

The online version of this article (10.1007/s00204-018-2250-8) contains supplementary material, which is available to authorized users.

## Introduction

Dopaminergic neurons account for less than 1% of neurons in the brain; their degeneration and loss leads to Parkinson’s disease (PD) (Chinta and Andersen 2006). It is estimated that, by 2030, 9 million individuals will be diagnosed with PD worldwide (Dorsey et al. 2007). While some known genetic factors play a role in the early onset of familial PD, monogenic forms only account for ~ 10% of patients. Around 90% of PD cases are sporadic, and represent the interplay of genetic risk and environmental factors (age and stress) or exposures (suspects include pesticides, flame retardants, metals, etc.) contributing to PD risk (Kalia and Lang 2015; Belin and Westerlund 2008). To better understand the risk posed by lifetime exposures, we must study how cells cope with toxicity and where the threshold of an effect lies. If cells can recover, it may be that underlying epigenetic changes or molecular scars alter the response to subsequent exposures. This presents questions as to whether cells are capable of recovering and when an adaptive response is sufficient to prevent adversity (Smirnova et al. 2015).

Complex I (NADH-ubiquinone oxidoreductase) of the electron transport chain is the molecular target for some compounds shown to induce PD-like symptoms [rotenone and MPP^+^, the active metabolite of 1-methyl-4-phenyl-1,2,3,6-tetrahydropyridine (MPTP)] in animals and humans (Sherer et al. 2007; Schapira et al. 1989; Parker et al. 2008). Other reported early cellular events include mitochondrial dysfunction (decreased ATP production and decreased membrane potential), oxidative stress, impaired proteostasis, and accumulation of misfolded proteins (https://aopwiki.org/aops/3) (Bal-Price et al. 2017a, b; Keane et al. 2011; Terron et al. 2018). Although dopaminergic degeneration has been studied extensively, recovery and adaptive mechanisms from toxicant exposure are rarely addressed. Currently, it is suggested that exposures throughout our lifetime as well as exposures earlier in life determine our risk for disease via gene–environment interactions. This could be studied in vitro by identifying how cells cope with single or repeated exposures.

Rotenone has been widely studied as one of the best-known dopaminergic toxicants and PD-inducing model compounds; it is extremely lipophilic, and freely crosses cellular membranes independently of any transporters. It has been shown to bind irreversibly to complex I of the respiratory chain (Lindahl and Öberg 1961; Grivennikova et al. 1997), and in vitro and in vivo studies have demonstrated that binding is necessary to reproduce PD mechanisms such as ROS accumulation (Sherer et al. 2003; Dhillon et al. 2008; Furlong et al. 2015). Moreover, dopaminergic neurons are more susceptible to rotenone toxicity than other neuronal cell types (Haddad and Nakamura 2015). Systemic rotenone exposure has become a widely used animal model of PD (Cannon et al. 2009; Daneshian et al. 2015). It is calculated that the chronic concentration required in the animal brain to induce dopaminergic degeneration is ~ 20–30 nM ‘free’ rotenone (Greenamyre et al. 2003). It has also been shown that rotenone can bind non-specifically to proteins other than complex I; therefore, it is considered that higher concentrations (> 30 nM) could have off-target effects (Higgins and Greenamyre 1996; Grefte et al. 2015). To study recovery and resilience, rotenone was selected, because there is copious literature reporting its acute toxicity in multiple in vitro models.

In vitro models to study neurotoxicity and dopaminergic toxicity in particular are needed as animal testing is demanding in terms of animal use, resources, and time. In vivo toxicity testing often shows inter-species differences, therefore, it is not always predictive of human health (Olson et al. 2000; Hartung 2011). The use of human cell lines can overcome these issues; however, the complexity of the central nervous system represents a major challenge for in vitro models. Current in vitro models (cancer cell lines, immortalized cell lines, primary cell cultures or stem cells) offer the advantage of a controlled environment to study molecular pathways involved in neurotoxicity (Hogberg et al. 2013; Falkenburger et al. 2016). Understanding the limitations of each model is important to determine whether it can answer the question being posed (Schmidt et al. 2017). Although iPSC-derived 3D models would be the most representative of the human brain due to their multicellular composition (Pamies et al. 2017), their complexity makes it difficult to attribute mechanisms to the respective cell type. Single-cell type models, differentiated in 3D, can, therefore, provide a tool to study cell-specific toxicant-induced disease mechanisms. It has been shown that many 3D cultures exhibit increased survival and enhanced neuronal differentiation compared to ones cultured in monolayer (Pamies and Hartung 2017; Smirnova et al. 2016; Alépée et al. 2014). The use of in vitro models allows us to study mechanisms by which environmental exposures lead to neurodegeneration, as well as neuroprotective pathways to identify biomarkers for the early diagnosis and therapy.

LUHMES (lund human mesencephalic) is a conditionally immortalized cell line, which overexpresses tetracycline-controlled *v-myc* (Lotharius et al. 2005; Scholz et al. 2011). These cells are suitable as a dopaminergic-cell model as they homogeneously differentiate, are electrically active and express functional dopamine transporter (DAT), vesicular monoamine transporter (VMAT-2), and the PD-related protein α-synuclein (ASYN) (Schildknecht et al. 2009). Furthermore, the LUHMES 3D model that we have developed can be kept in culture for up to 21 days, and is suitable for long-term and wash-out studies. The primary advantage of using a 3D model for this study is that aggregates are cultured in suspension; therefore, compounds that easily bind to plastic such as rotenone can be washed out more effectively than in monolayer cell models (Smirnova et al. 2016; Harris et al. 2017).

Our question, which has yet to be addressed to a greater extent in in vitro toxicology, is cellular recovery and resilience to toxic insult (Smirnova et al. 2015). Cellular resilience is a complex cellular mechanism, which, to date, has been mostly studied within infectious diseases (Richardson 2016) as well as neuroprotection after trauma and plasticity (Osório et al. 2017; Arenaza-Urquijo and Vemuri 2018). Some recent studies address neuronal processes of reverting “back to normal” and reversal of apoptosis (“anastasis”) (Manji et al. 2000; Tyagi et al. 2015; Pfau and Russo 2015). Our hypothesis is that cells can overcome low-dose toxicant effects (in which cell death is not triggered), and then, become either resilient or more susceptible to subsequent exposures (via activation of cell survival/death pathways, changes in gene expression, or epigenetic modulations) (Smirnova et al. 2015). One hypothesis that has yet not been tested is whether resilience mechanisms are beneficial or detrimental to cells in the long-term as permanent activation or inhibition of specific pathways may contribute to disease pathology (Daskalakis et al. 2013; Pfau and Russo 2015). In neurodegenerative diseases, the final steps of disease manifestation have been well characterized in human post-mortem samples and in vivo studies. However, the early mechanisms linking environmental exposures to disease are still unknown and are becoming more relevant to understanding long-term adverse outcomes. The 3D LUHMES model can be applied to study susceptibility to subsequent exposures as well as molecular memory to the previous exposures as shown here. Although some in vitro studies have focused on low-dose, chronic exposures to toxicants showing long-term lesions (Sherer et al. 2002; Drolet et al. 2009; Borland et al. 2008), recovery and resilience to dopaminergic neurotoxicity have not been previously shown.

## Materials and methods

A detailed description of materials and methods can be found in Supplementary Methods.

### LUHMES 3D culture and differentiation

LUHMES (ATCC^®^ CRL_2927™) 3D cell culture and differentiation protocol were followed as described (Harris et al. 2017). Briefly, cells were used between passages 15 and 25. 4 × 10^6^ cells were placed in a 175 cm^2^ flask for 48 h to expand cells. On day 0, 3D-differentiation was initiated: 5.5 × 10^5^ cells were seeded into each well of a 6-well plate and placed on a gyratory shaker at 80 rpm (50 mm orbit diameter) in an incubator at 37 °C, 10% CO_2_, and 95% humidity.

### Toxicant treatment and wash-out

To study delayed effects of toxicant treatment and resilience, aggregates were exposed to rotenone or DMSO (vehicle control) for 24 h with subsequent wash-out of these compounds. A concentration of 100 nM rotenone was chosen for treatment as this concentration represented the lowest observed adverse effect level (LOAEL) for viability in 2D and 3D LUHMES (previously reported in Krug et al. 2014; Smirnova et al. 2016). Since gene expression is considered as one of the most sensitive endpoints where alterations can occur before any visible changes in functionality and viability, a lower concentration of 50 nM was included for microarray analysis and resilience experiments. Rotenone was dissolved in 100% DMSO at a stock concentration of 100 mM (aliquoted and stored at − 20 °C). Experiments were performed in 6-well plates. Aggregates were treated with 100 nM rotenone on day 7 of differentiation. 24 h later; on day 8, half of the samples were collected, and, in the remaining cultures, rotenone was washed out as described before (Harris et al. 2017). Importantly, in the wash-out experiments, the aggregates were transferred to new cell culture plates by bringing them to the center of the well using circular motions and pipetting them in a 100 µL volume into a new well containing 2 mL fresh medium to effectively remove exposure to rotenone, which might stick to the plastic, as described thoroughly in Harris et al. (2017). Medium was changed every other day up to day 15.

### Rotenone quantification in medium by mass spectroscopy

Three types of samples were produced: (I) 100 nM rotenone in cell culture medium in low-absorbance vessels; (II) 100 nM rotenone in a standard coated cell culture dish; (III) 100 nM rotenone in a coated cell culture dish containing cells. Rotenone content was measured from conditions II and III after 24 h or after wash-out and 7 day recovery period (day 15). For condition III, cells were cultured exactly as described under LUHMES 3D culture and differentiation. Rotenone was extracted from the samples with methyl-, *tert*-butyl-ether (MTBE). From each sample, 40 µL were transferred into a fresh vial and an equal volume of water containing 0.1% formic acid (Fisher Scientific) was added. The sample was transferred to a supported liquid extraction plate. A positive pressure was applied to load the sample into the plate. MTBE (Fisher Scientific) was added to each well and left to pass through the plate and into a glass insert. The collected samples were dried under nitrogen at 30 °C. Each sample was reconstituted in a volume of MTBE and capped for injection into LC–MS (Waters Xevo QTof G2-S, Agilent 7890B, CTC PAL LHX-xt autosampler). Mass spectrometric conditions (Corona Discharge: 3 µA, Polarity: positive ion, Cone gas: 100 L/h, Aux gas: 140 L/h, Lockmass: Heptacosa, and MS: positive ion, *m*/*z* 50–800 in 0.2 s). To quantify the rotenone amount, a serial dilution of solution I was quantified to obtain a calibration curve. Rotenone content of wells with and without cells was normalized to this calibration sample (considered as 100%). The rotenone amount bound to plastic was calculated from the difference between samples I–II. Intracellular rotenone amounts were calculated from the difference II–III. The experiment was performed 6–8 times.

### Viability assays

Resazurin assay was performed as described in Harris et al. (2017). Experiments were performed in three independent experiments with technical triplicates. LDH was measured in the medium in control and treated samples following the manufacturer’s instructions (CytoTox 96^®^ NonRadioactive Cytotoxicity Assay, Promega).

### DNA quantification

Aggregates were lysed and DNA extracted using phenol:chloroform:isoamyl (24:25:1, Sigma) extraction. DNA quantification was performed using the Qubit dsDNA Broad Range Assay Kit (Invitrogen) and Qubit 2.0 Fluorometer (Invitrogen) according to the manufacturer’s instructions.

### RNA extraction, reverse transcription, and real-time PCR

Total RNA was extracted using either TRIzol^®^ Reagent (Life Technologies) followed by RNA Clean and Concentrator™-Kit (Zymo Research^®^) or mirVana microRNA isolation kit (for microarray analysis) following the manufacturer’s instructions. Detailed description of cDNA synthesis and PCR is described in Supplementary Methods. Primers used for PCR are listed in Supplemental Table S1.

### Microarray analysis

Microarray analysis was conducted at The Johns Hopkins Bloomberg School of Public Health Genomic Analysis and Sequencing Core Facility. RNA was extracted from three samples per condition of LUHMES aggregates on days 8 and 15 using the mirVana miRNA Isolation kit (Ambion/Thermo Fisher Scientific) according to the manufacturer’s protocol. Following elution of purified RNA from the mirVana miRNA columns with nuclease-free water with RNasin, quantitation was performed using a NanoDrop spectrophotometer and quality assessment determined by RNA LabChip analysis on an Agilent BioAnalyzer 2100 or RNA Screen tape on an Agilent TapeStation 2200.

One hundred nanograms of total RNA were processed for hybridization to Agilent SurePrint G3 Human Gene Expression v2 8 × 60K Arrays according to Agilent’s One-Color Microarray-Based Analysis (Low Input QuickAmp Labeling) protocol, including cRNA synthesis with Cy3-labeling and purification, fragmentation, hybridization, and washing. Spike-in controls were utilized and processed according to Agilent’s One-Color RNA Spike-In kit protocol.

The arrays were scanned in the Agilent G2600D SureScan Microarray Scanner using scan protocol AgilentG3_GX_1color for gene expression arrays. Agilent’s Feature Extraction Software Version 11.5.1.1 was used to assign grids, provide raw image files per array, and generate QC metric reports from the microarray scan data. The QC metric reports were used for quality assessment of all hybridizations and scans.

Txt-files from Feature Extraction Software were exported for further analysis with R version 3.4.2 (https://www.R-project.org/) and Bioconductor version 3.6 (Huber et al. 2015; Gentleman et al. 2004). In a first step, the arrays gMedianSignal were imported, normexp background corrected (Ritchie et al. 2007; Silver et al. 2009) and quantile normalized between arrays (Bolstad et al. 2003). Primary QC by principal components analysis revealed a batch effect on a subset of arrays, which were processed at a different time-point with a different washing procedure, which was corrected by parametric empirical Bayes frameworks for adjusting data for batch effects as implemented in ComBat (Johnson et al. 2007).

Probes were then filtered out if they are not least 10% brighter than the 95% percentile of the negative control probes on each array on at least three arrays (original 62,976 probes, after 54,135 probes). In a next step, control probes were filtered out (after 51,849 probes) and duplicate probes were summarized (after 44,414 probes). Individual probes which were either labeled by the Agilent Feature Extractor Software as not to be used, Non-uniform outlier or Population outlier were removed, as well (174 probes over all arrays). In the last step, probes, which did not map to Entrez-ID, were removed (32,123 probes left) and probes were averaged per Entrez-ID (22,150 unique Entrez-IDs left). Differential expression was estimated by empirical Bayes moderation of the standard errors towards a common value (Empirical Bayes moderated *t* test) (Smyth 2004). The transcriptomics microarray data sets have been deposited in the Gene Expression Omnibus (GEO, GSE116280). Over-representation analysis was done on all genes with FC > 1.5 and p(FDR) < 0.05 for the day 8 data set and on FC > 1.5 and p (not adjusted) < 0.05 for the day 15 data set with MSigDB C2 and C3 gene sets, with an FDR < 0.05 for significant annotations.

### Complex I activity assay

Mitochondria Isolation was performed on ice using the reagent-based method (Mitochondria Isolation Kit for Tissue and Cultured Cells, BioVision); Complex I activity using mitochondrial Complex I Activity Colorimetric Assay Kit (BioVision) following the manufacturer’s instructions (see Supplementary Materials for details). This kit uses decylubiquinone, an analog of ubiquinone, as an electron acceptor that gets converted to decylubiquinol through the catalytic activity of Complex I. The Complex I dye absorbs light at 600 nm in its oxidized form and is used as a terminal electron acceptor that accepts electrons from decylubiquinol. Complex I activity is determined colorimetrically by recording the change in absorbance of reduced Complex I dye at 600 nm. Activity was measured in three independent experiments in technical duplicates.

### ATP assay

The bioluminescence ATP Assay Kit (Thermo Fisher Scientific, A22066) was used to determine the amount of intracellular ATP in aggregates according to the manufacturer’s instructions. Although this is also a measure of viability, in contrast to the resazurin assay which measures mitochondrial metabolic activity, this endpoint was included to determine total ATP levels produced from aerobic and anaerobic respiration in viable cells. The resazurin assay relies on the assumption that there is an equal cell/aggregate number per well, which is not necessarily the case after multiple washing steps. The ATP assay allows for normalization to total protein content and, therefore, more accurately represents perturbations to energy metabolism in viable cells (see Supplementary Materials for details). Average luminescence values ± SEM were calculated from at least four independent experiments with technical triplicates.

### Electron microscopy and mitochondria quantification

Reagents were bought from Electron Microscopy Sciences (Fort Washington). 3D LUHMES aggregates were fixed using a solution of 3.0% formaldehyde, 1.5% glutaraldehyde contained in 100 mM sodium cacodylate, 5 mM Ca^2+^, and 2.5% sucrose at pH 7.4 for 1 h at room temperature. Subsequently, samples were washed three times for 15 min using a solution of 100 mM cacodylate containing 2.5% sucrose at pH 7.4; post-fixed with Palade’s OsO_4_ at 4 °C; and rinsed 1× using Kellenberger UA (uranyl acetate) and left in UA at RT overnight in the dark. Samples were then dehydrated through a graded series of ethanol (50, 70, 95, and 100%) at 4 °C; followed by three 15 min washes in fresh 100% ethanol at RT. Following, two 5 min exchanges with propylene oxide (PO), samples were placed in a mixture of 50:50 Epon/propylene oxide and left overnight, uncovered, under vacuum. The resin mixture was replaced with fresh 100% Epon and left under vacuum an additional 4–6 h, and, subsequently, polymerized in an oven at 60 °C for 24–48 h. 80 nm sections were then cut on a Leica UCT ultramicrotome and placed on 400 mesh copper grids. Samples were imaged using a Philips EM 420 transmission electron microscope. Images were collected with a Megaview III side-entry camera from Olympus Soft Imaging Systems (OSIS); and mitochondrial area and diameter assayed using iTEM software (also available from OSIS). Quantification was performed by selecting 20 random images from either vehicle control or treated, ranging from low (3300×) to high (31,000×) magnification in three independent experiments. The square area was measured for the entire image excluding the grid bars, when present. The mitochondria in each image were counted; and discrimination of healthy vs. unhealthy was assessed based on the appearance of the mitochondrial matrix density. Using a straight-line measurement tool, the length from the narrowest part of the mitochondria was measured and used as the diameter.

### Neurite outgrowth imaging and analysis

Red fluorescent protein (RFP)-expressing LUHMES (Schildknecht et al. 2013) were differentiated and treated as described above. On day 8 or day 15, aggregates were seeded on Matrigel™ (BD Biosciences) pre-coated, flat-bottom, black 24- or 96-well plates (Thermo Fisher Scientific). After 24 h, wells were fixed in 4% PFA and imaged using a confocal microscope (with open pinhole) and analyzed using the Sholl Image J Software (https://imagej.net/Sholl_Analysis). To analyze this data, the ratio was calculated for each shell (number of intersections/distance from aggregate) and the mean plotted. Curves were compared using a quadratic non-linear regression fit with confidence intervals.

### Electrical activity

Whole-cell recordings were performed under a DIC microscope (eclipse E600FN, Nikon). 3D LUHMES were transferred to the recording chamber with culture media at 37 °C. Every 30 min, the media were replaced. To target whole-cell recordings, 3D LUHMES aggregates were attached to a glass pipette by means of a gentle negative pressure, which was released once the aggregate was attached. Cells were visualized at high magnification (40× objective, water immersion) and chosen with respect to their morphological phenotype (small, round, phase-bright cell bodies). Patch pipettes (4–5 MΩ resistance) made of borosilicate glass were filled with an internal solution containing 130 mM K-gluconate, 10 mM KCl, 0.2 mM EGTA, 10 mM HEPES, 0.5 mM Na3GTP, 4 mM MgATP, and 10 mM Na-Phosphocreatine (pH adjusted to 7.3 with KOH, 285–295 mOsm). Once stable, whole-cell recordings were performed and basic electrophysiological properties were examined through depolarizing current injections. Electrophysiological data were acquired with a Multiclamp 700A amplifier (Molecular Devices), data acquisition board (model PCI MIO 16E-4, National Instruments), and Igor Pro (Wavemetrics). Data were filtered at 4 kHz and digitized at 10 kHz. Minimal spike latency was measured using a single exponential fit for the spike latency vs. the current injection strengths. Differences in treated and control samples were analyzed for statistical significance using Mann–Whitney *U* test.

## Results

### The LUHMES 3D cell model is suitable for recovery and resilience experiments

**Fig. 1:**
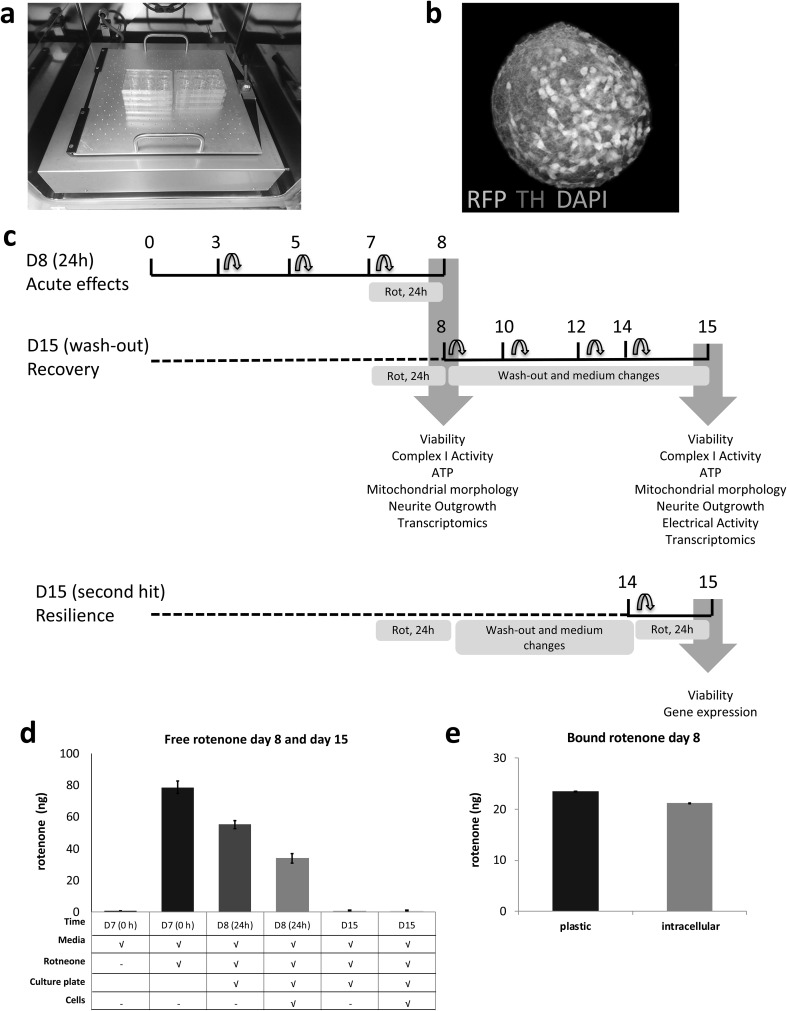
LUHMES 3D model for acute, recovery, and resilience experiments. **a** LUHMES differentiated in 3D on a gyratory shaker showing **b** RFP-expressing cells (red) and TH (green), nuclei (blue). **c** LUHMES 3D treatment and wash-out scheme for recovery and resilience (second hit) experiments and endpoints. **d** Medium rotenone quantification prior to treatment, day 8 and day 15. From left to right, bars correspond to negative control (medium without rotenone), positive control (medium with rotenone prior to treatment), 24 h treatment control (medium with rotenone in plates), 24 h treatment (medium with rotenone in plates with aggregates), 7 day wash-out control (medium with rotenone in plates on day 15 after wash-out), and 7 day wash-out treated cells (medium with rotenone in plates on day 15 after wash-out with aggregates). **e** Amount of rotenone bound to plastic and cells after 24 h exposure (day 8). (Color figure online)

In this study, we used the previously developed and characterized 3D LUHMES in vitro model (Smirnova et al. 2016) (Fig. 1). Cells (5.5 × 10^5^) were seeded into each well of a 6-well plate, forming ~ 200 aggregates/well (250–300 µm in diameter), over 15 days of differentiation. By quantifying DNA, each aggregate was calculated to be composed of 3000–5000 cells (Supplemental Figure S1). To determine whether LUHMES 3D in vitro cultures can recover from low-dose rotenone effects, we followed the same treatment protocol as previously reported (Harris et al. 2017; Smirnova et al. 2016) focusing on 24 h exposure to 100 nM rotenone. In addition to (1) acute cellular response on day 8 of differentiation (D8 24 h), we studied (2) delayed response and recovery on day 15 after compound wash-out and additional 7 days in culture (D15 wash-out); (3) cellular response to a second-rotenone exposure after recovery on day 15 (D15 R100-R100). The treatment scheme for these conditions is depicted in Fig. 1. A key advantage of the 3D culture is that, in the wash-out experiments, the aggregates can be transferred to clean cell culture plastic ware to ensure that the highly lipophilic rotenone is removed by washing without destroying cells and neurites which is currently not possible in traditional monolayer cultures. Using mass spectroscopy, we quantified free rotenone in the medium before treatment, 24 h after treatment, and 7 days after wash-out. These results demonstrated a decrease in free rotenone in medium after 24 h, when 78.8 ng (100 nM) were added to cell culture plates, meaning that 23.5 ng were bound to plastic (Fig. 1d (bar II minus bar III), e). Furthermore, in the presence of cells, a further decrease in free rotenone was recorded; demonstrating that 21.1 ng were bound to cells within aggregates (Fig. 1d (bar III minus bar IV), e). No free rotenone was detected in medium on day 15 after wash-out (bar V and VI, Fig. 1d).

**Fig. 2:**
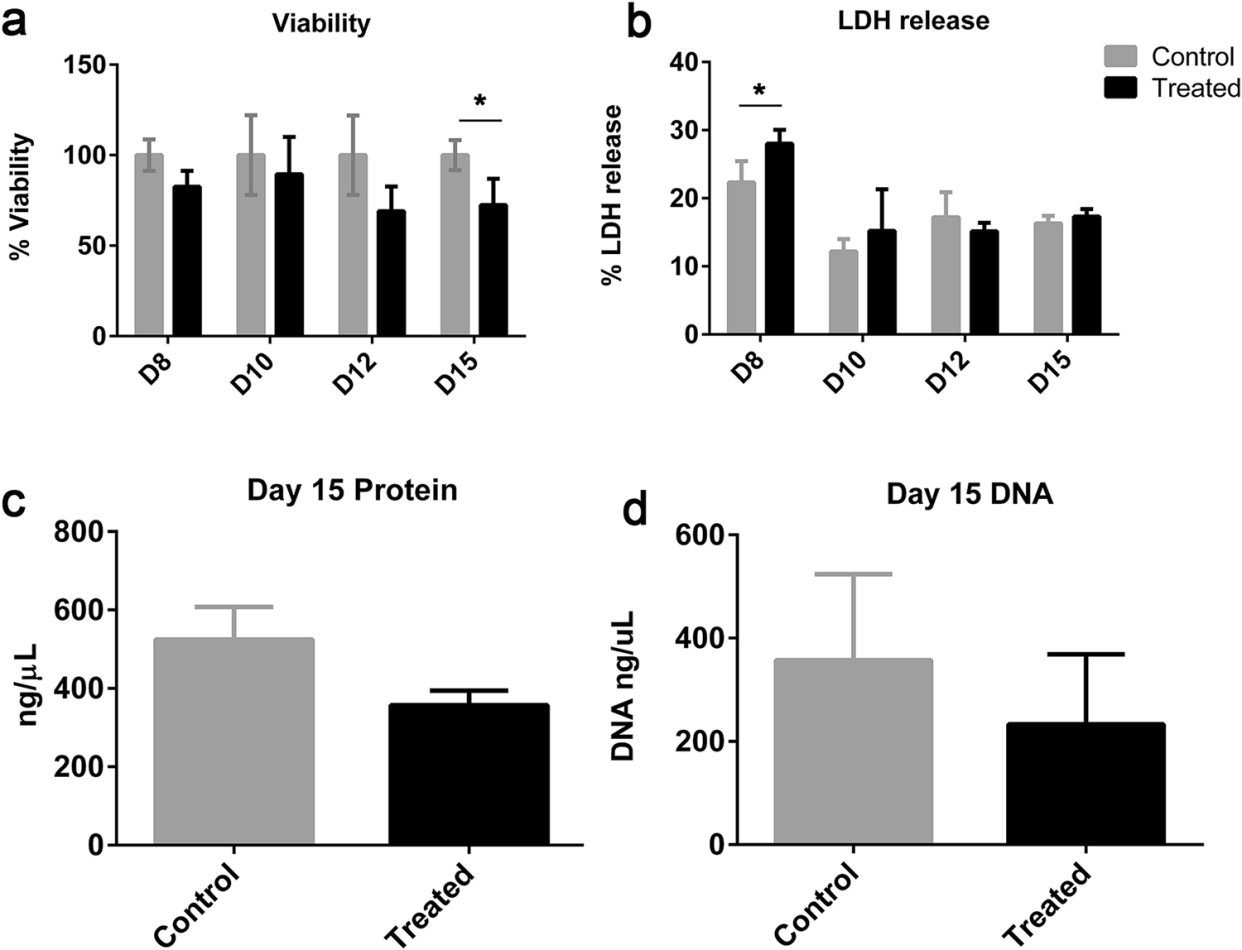
3D LUHMES viability after wash-out. **a** Cell viability measured over time using resazurin assay on days 8 (after 24 h treatment) and 10, 12, and 15 (throughout recovery). **b** Cytotoxicity over time during recovery measured by LDH release on days 8 (after 24 h treatment) and 10, 12, and 15 (throughout recovery). **c** Protein concentration on day 15 after wash-out and 7 day recovery. **d** DNA quantification on day 15, after wash-out and 7 day recovery. All data were normalized to untreated control cells and are displayed as means ± SEM from three independent experiments. *p < 0.05

In the previous work, we have shown that 100 nM rotenone produces ~ 15% decrease in viability (as measured by resazurin reduction assay) after 24 h exposure (day 8) and further 10–15% reduction until day 15 after compound wash-out (Smirnova et al. 2016; Fig. 2a). The acute effect on cell viability has been shown also in other in vitro cultures (Sherer et al. 2003; Krug et al. 2014). To define when cytotoxicity occurs between days 8 and 15, we measured viability and LDH release every 48 h. The viability level on day 10 was similar to day 8. We identified that the further 10% decrease in viability occurs between days 12 and 15 (Fig. 2a). In addition, we observed a small but a significant increase in LDH release on day 8 (Fig. 2b). However, from days 10 to 15, no significant increase in LDH was observed compared to controls. This indicated that the remaining cells could recover from the short-term rotenone exposure.

Since the resazurin assay specifically detects cellular metabolic activity, we studied whether the observed reduction was due to a decrease in metabolic activity or due to a lower cell number in rotenone-treated samples. For this reason, we measured DNA content (surrogate for cell number) and protein concentration, as a true measure of sphere viability, after rotenone wash-out and 7 day recovery. Both, DNA and protein levels were lower indicating some cells were gradually lost after wash-out and recovery period (Fig. 2c, d).

### ATP levels are increased and mitochondria recover after rotenone wash-out

**Fig. 3:**
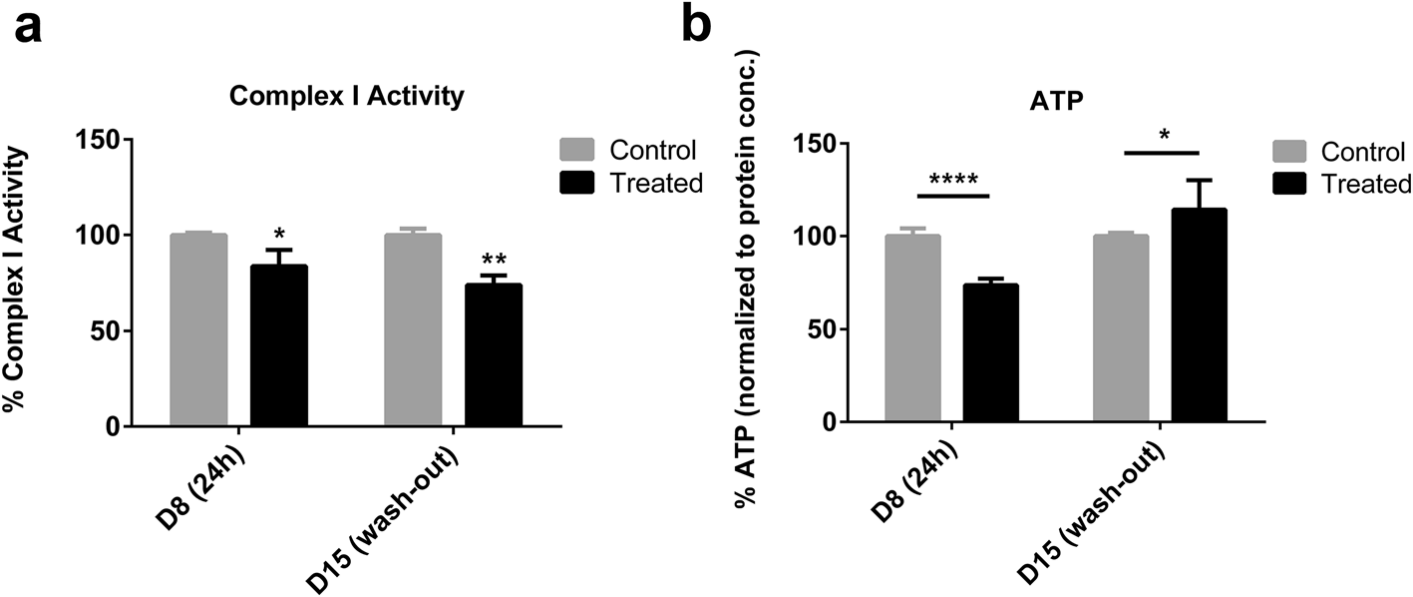
Effects of rotenone on complex I activity and ATP levels. **a** Complex I activity after rotenone exposure (day 8) and after compound wash-out and recovery (day 15) in control and treated samples. **b** ATP levels after rotenone exposure (24 h, day 8) or after wash-out and 7 day recovery period (day 15) in control and treated samples. Differences in treated and control samples from at least three independent experiments were analyzed for statistical significance using unpaired Student’s *t* test. A *p* value < 0.05 is denoted on graphs by * and *p* < 0.0001 by ****, respectively

Rotenone is a known complex I inhibitor which decreases ATP production (Sherer et al. 2003). To better understand changes occurring at a molecular level in the remaining survived cells, we assessed complex I activity and ATP levels after 24 h exposure to 100 nM rotenone [D8 (24 h)] and 7 days after wash-out [D15 (wash-out)]. Mitochondria were extracted from control and treated samples, complex I activity was measured in samples using a colorimetric assay and normalized to protein content. As expected, complex I activity was reduced after 24 h rotenone treatment; and remained inhibited after wash-out (Fig. 3a). ATP was measured in cell lysates using a luminescence assay (normalized to protein levels). In concordance with earlier acute in vitro reports (Sherer et al. 2003), a significant decrease in ATP levels (~ 30%) was observed after 24 h exposure (Fig. 3b). However, on day 15 (after wash-out and recovery period), ATP levels were significantly increased in rotenone-treated samples compared to controls, despite the decreased cell number and ~ 20% inhibition of complex I activity (Fig. 3b). Importantly, ATP levels and complex I activity were normalized to protein content. Thus, although there was a decrease in number of viable cells on day 15 after wash-out (Fig. 2), the remaining cells had increased ATP levels compared to vehicle-treated control samples.

**Fig. 4:**
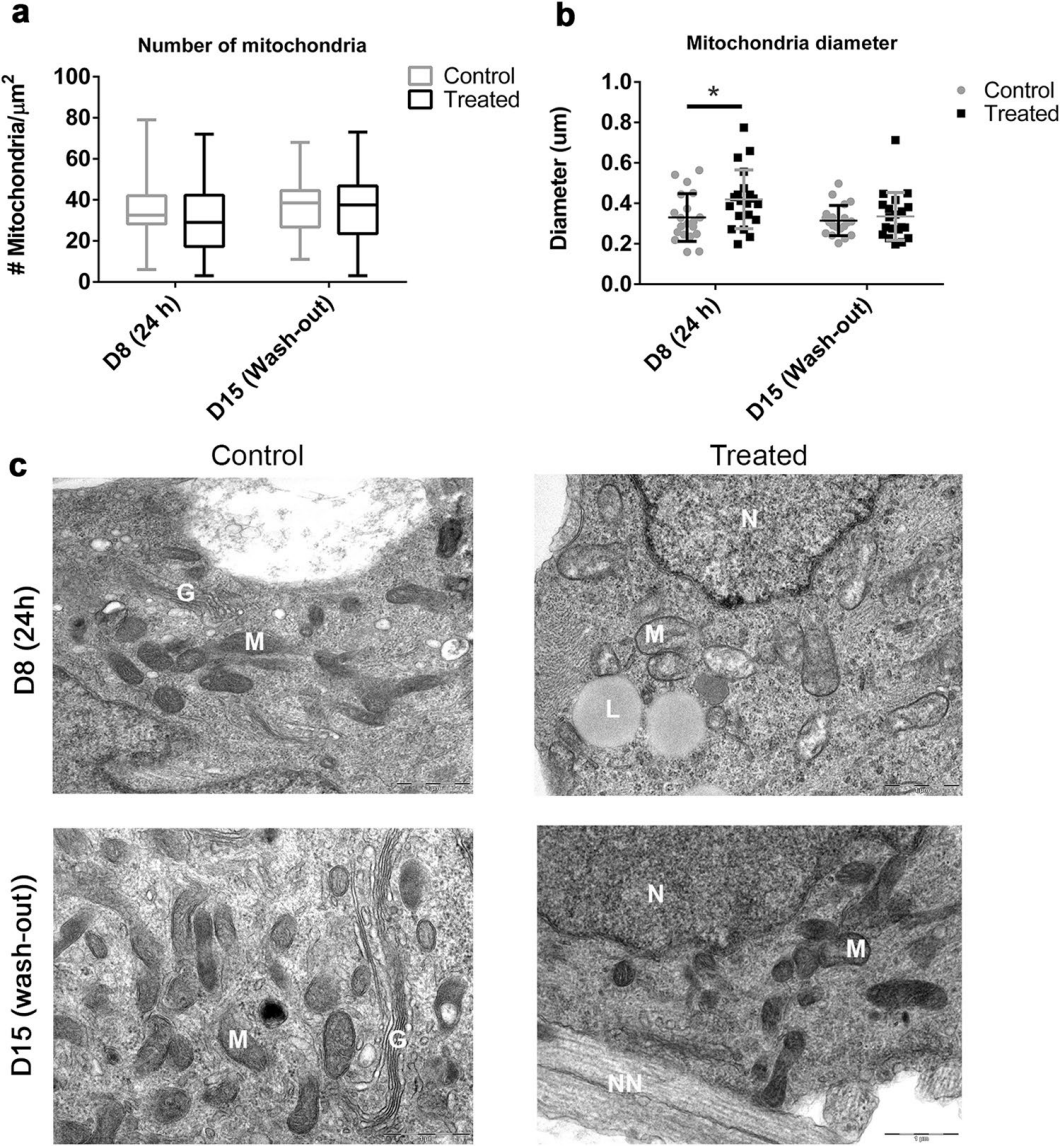
TEM analysis of mitochondria after rotenone exposure and wash-out. *M* mitochondria, *G* Golgi complex, *L* lipid droplets, *N* nucleus, *NN* neurite. The number (**a**) and diameter (**b**) of mitochondria from random image areas were quantified on day 8 (24 h) and day 15 (wash-out). Data from 20 random images from three independent experiments are shown as well as means ± SD. Differences between treated and untreated samples were analyzed for statistical significance using unpaired Student’s *t* test. A *p* value < 0.05 is denoted on the graphs by asterisk. **c** Representative images are shown with arrows indicating morphological alternations to the mitochondrial membrane

As the observed effects on ATP levels could not be explained by changes in cell number, we examined mitochondria physiology by electron microscopy. There was no change in the number of mitochondria (Fig. 4a). Mitochondrial diameter was recorded to investigate whether shrinking or swelling was taking place after treatment and wash-out. After 24 h rotenone exposure, mitochondrial diameter was increased by ~ 27% on average (Fig. 4b, c, day 8 control avg = 0.33 µm; day 8 treated avg = 0.42 µm, *p = 0.0378). After compound wash-out and 7 day recovery, mitochondria diameter was comparable to controls.

In summary, these data suggest that the cells remaining in the aggregates after acute exposure and the recovery period were able to compensate for complex I inhibition, recover ATP production, and restore mitochondria morphology.

### Neurons recover neurite outgrowth after rotenone wash-out and are electrically active

**Fig. 5:**
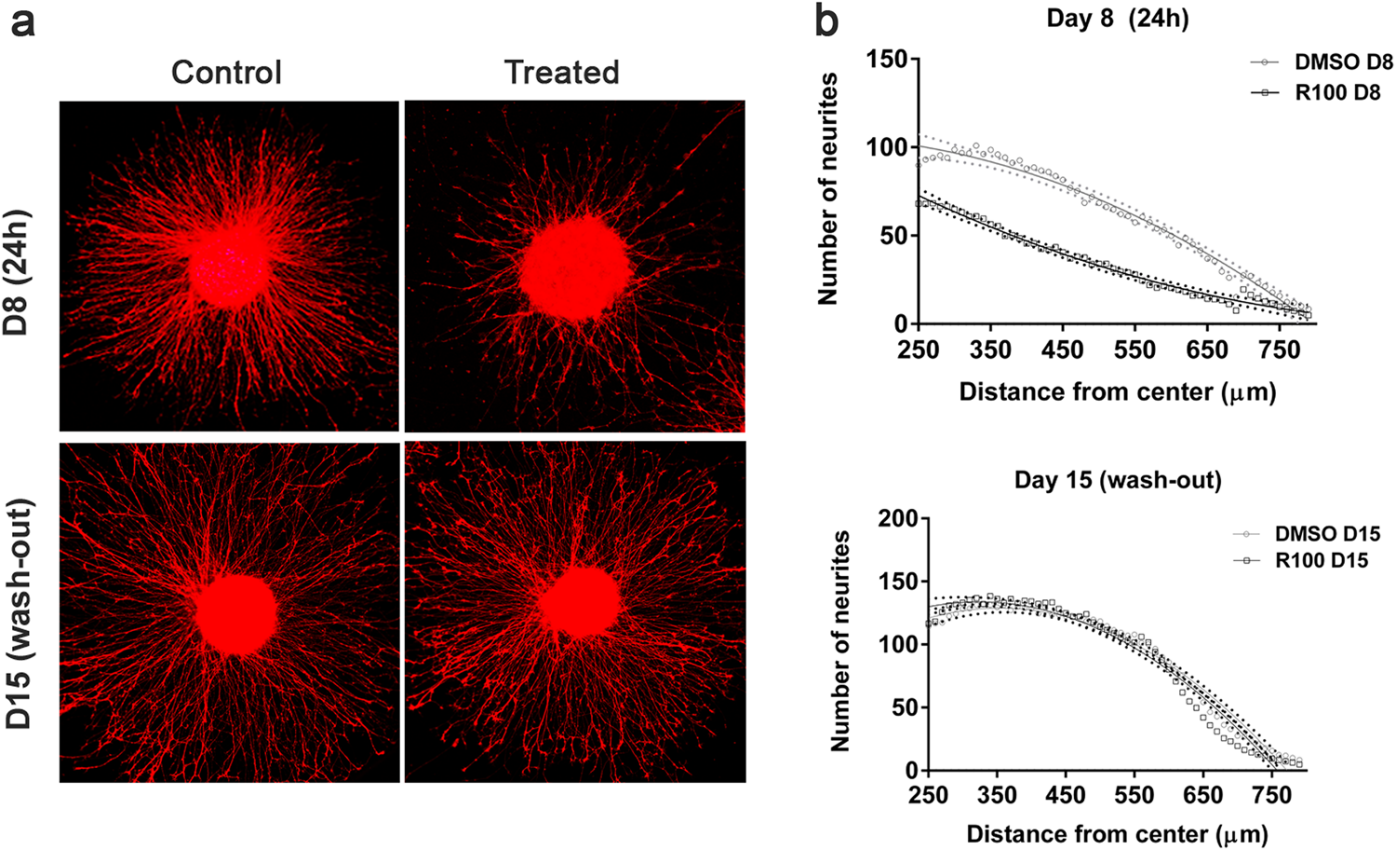
Image J Sholl analysis of neurite outgrowth after rotenone exposure (day 8) and wash-out (day 15). RFP-LUHMES aggregates were grown on Matrigel^®^ on day 8 or day 15. **a** Representative images for the different conditions are shown. **b** Sholl analysis (Image J) was used to calculate the number of neurites at different distances from the aggregate center on day 8 and day 15 from three independent experiments (5 individual aggregates per experiment). Curves were compared using a quadratic non-linear regression fit with confidence intervals

Next, we analyzed whether the remaining viable neurons are fully functional after the recovery period. We tested whether cells are still able to prolong their neurites when given space and an appropriate stimulus. For this reason, the aggregates were plated on Matrigel^®^, a condition favoring neurite outgrowth from aggregates. Aggregates were plated either immediately after rotenone treatment [D8 (24 h)] or after a 7 day recovery period [D15 (wash-out)] and outgrowth was quantified using the Image J Sholl image analysis (Fig. 5; Figure S2). Our results show that acute exposure (100 nM, 24 h) decreased the number and length of neurites (R100 D8 slope = − 0.1226 ± 0.003 vs. DMSO D8 slope = − 0.1822 ± 0.006). Aggregates plated after the 7 day recovery period, which showed no differences in number or length when compared to control samples (R100 D15 slope = − 0.245 ± 0.015 vs. DMSO D15 slope = − 0.279 ± 0.016) (Fig. 5b).

**Fig. 6:**
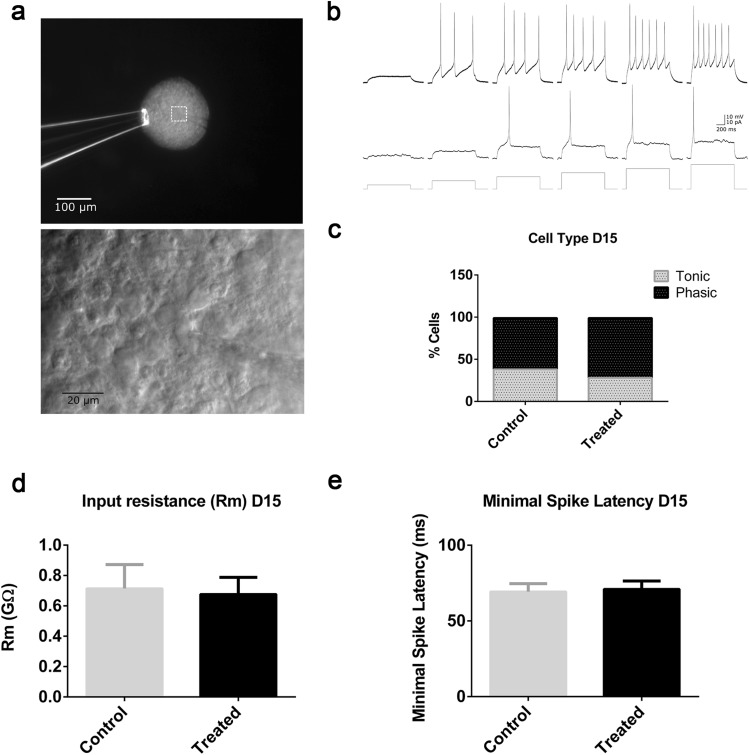
3D LUHMES electrical activity on day 15 after acute exposure on day 7 and compound wash-out. **a** Photo microscopy image of a 3D LUHMES aggregate attached to a glass pipette and a patched cell at a higher magnification. Cells on different aggregates were patched in three independent experiments. **b** Firing pattern of a representative tonic (top) and a phasic (middle) cell with voltage responses to 1 s current injections (bottom) at 4, 8, 12, 16, 20, and 24 pA. **c** Total number of tonic and phasic cells in control and treated samples on day 15 (*p* = 0.695 two-sided Fisher’s exact test); **d** input resistance (*R*_m_) of the phasic cells (*p* = 0.963 two-sided Mann–Whitney *U* test) and **e** minimal spiking latency of phasic cells (*p* = 0.852 two-sided Mann–Whitney *U* test). Error bars represent SEM

LUHMES monolayer cultures have been shown to be electrically active (Scholz et al. 2011). As a further functional endpoint, we used whole-cell patch clamp recording (Fig. 6a) to evaluate whether (1) LUHMES 3D culture contains electrically active cells on day 15 of differentiation, and (2) is electrical activity affected on day 15 after treatment on day 7 and 7 day recovery. Both tonic and phasic modes of activity were identified in LUHMES aggregate cells (Fig. 6b). On day 15, no differences were observed in the number of tonic vs. phasic cell types (Fig. 6c). We focused on the physiological properties of phasic cells. No changes in the input resistance (Fig. 6d) or spike latency (Fig. 6e) were detected in phasic neurons after recovery. This demonstrated that there were no delayed effects of rotenone on electrical activity in measured cells.

In summary, these data together suggest that cells are functional after rotenone wash-out and the recovery period.

### Acute transcriptomic changes are overcome after recovery period

**Fig. 7:**
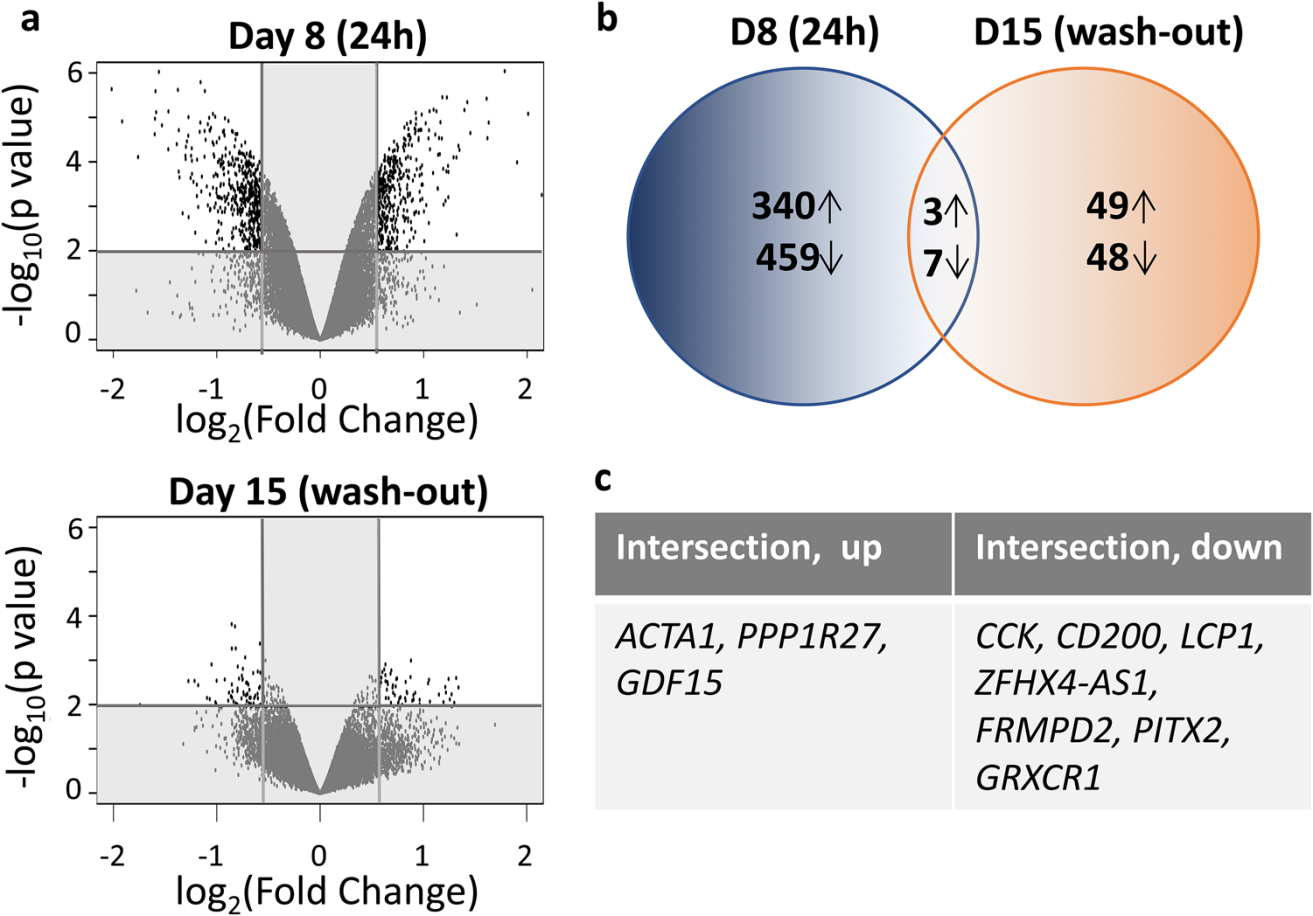
Rotenone-induced transcriptome changes on day 8 (24 h) vs. day 15 (wash-out). **a** Volcano plots show the global changes in transcriptome for day 8 and day 15. **b** Venn diagram shows the number of up- and downregulated genes on day 8 [D8 (24 h)] and on day 15 [D15 (wash-out)] (FC > 1.5, *p* < 0.01). Ten genes were in intersection between two conditions, which are listed in **c**. For this diagram, the *p* values were not adjusted for multiple testing. *ACTA1*actin alfa 1, skeletal muscles, *PPP1R27* protein phosphatase 1, regulatory subunit 27, *GDF15* growth differentiation factor 15, *CCK* cholecystokinin, *CD200* OX-2 membrane glycoprotein, *LCP1* plastin 2 (lymphocyte cytosolic protein 1), *ZFHX4* AS1-ZFHX4 (Zinc-Finger Homeobox 4) antisense RNA 1, *FRMPD2* FERM and PDZ domain containing 2, *FRMPD2* FERM and PDZ domain containing 2, *GRXCR1* glutaredoxin and cysteine-rich domain containing 1

We further analyzed the effects of 100 nM rotenone exposure on the LUHMES aggregates transcriptome 24 h after treatment [D8 (24 h)] and after compound wash-out and 7-day recovery period [D15 (wash-out)]. We found 708 genes significantly changed on day 8 (FC > ± 1.5 and *p* (adjusted) < 0.05). On day 15, after multiple hypothesis testing correction, no significantly changed genes remained. Since we performed low-dose short-term exposure and compound wash-out, we did not expect dramatic changes in gene expression on day 15, especially considering that the functional endpoints, described above, indicated recovery. However, previously, we could observe some slight changes in gene expression on day 15 by qPCR (Smirnova et al. 2016). In addition, because qPCR is more sensitive than microarray method and the FDR correction of a big data set (over 20,000 genes) with a small sample size (three replicates per condition) may hide slight but still significant changes, we used unadjusted *p* values for further analysis. To be more stringent, we decreased the p value cutoff for all microarray analysis (*p* < 0.01 vs. classically used *p* < 0.05). On day 8, 809 genes were significantly changed, with 343 upregulated and 466 downregulated genes (Supplemental Table S2). On day 15, a significantly lower number of genes were perturbed (107, FC > ± 1.5, *p* < 0.01) with 52 up- and 55 downregulated genes (Fig. 7a, b; Supplemental Table S3), There were ten genes in the intersection of day 8 and day 15 (Fig. 7b, c). The same analysis was performed for samples exposed to 50 nM for 12 or 24 h showing less of an effect with the lower concentration and shorter exposure time, as expected (Supplemental Tables S4–S7 and Figure S3).

The genes from day 8 (R100, 24 h) with an FDR corrected p value of less than 0.05 (1516 genes total) as well as the genes from day 15 with an uncorrected *p* value of 0.05 (1092) genes were examined for enrichment analysis; samples were highly enriched for genes related to neurogenesis as well as genes associate with Alzheimer’s on day 8 (Supplemental Table S8). After wash-out and recovery on day 15, samples were enriched for neurogenesis as well as plasma membrane components, and CNS development (Supplemental Table S9). In addition, both gene sets from day 8 and day 15 were explored for potential interactions via the STRING database; both were significantly enriched for known protein interactions. The resulting network from day 8 had *TOP2B* as the main “hub” (most highly connected protein). The switch from *TOP2A* to *TOP2B* is known to be critical for neuronal differentiation in vitro and in vivo; In addition, *TOP2B* appears to selectively occupy regulatory regions in the genome where it modulates the transcription of genes involved in neuronal survival (Tiwari et al. 2012) (TOP2B subnetwork, Supplemental Figure S4a.)

Of the genes in common between day 8 and day 15, *CCK* was consistently highly connected (i.e., a “hub”) in both interaction networks. The role of *CCK* in the brain is poorly understood, however, in this data set, the subnetwork of *CCK* for day 8 and day 15 were both highly enriched for genes on the pathway for non-odorant GPCR (GPCR, class A, Rhodopsin-like), (corrected *p* value of 2.072 × 10^−15^ day 8.884 × 10^−6^ day 15) (day 8 Supplemental Figure S4b, day 15 Supplemental Figure S4c). Given the relatively weak signal from the transcriptomics data from day 15, it is difficult to draw firm conclusions, but the data suggest that there is a persistent alteration in non-odorant g-protein-coupled receptors mediated in part by *CCK* signaling.

### Metabolic resilience is observed with the second exposure to rotenone after recovery

**Fig. 8:**
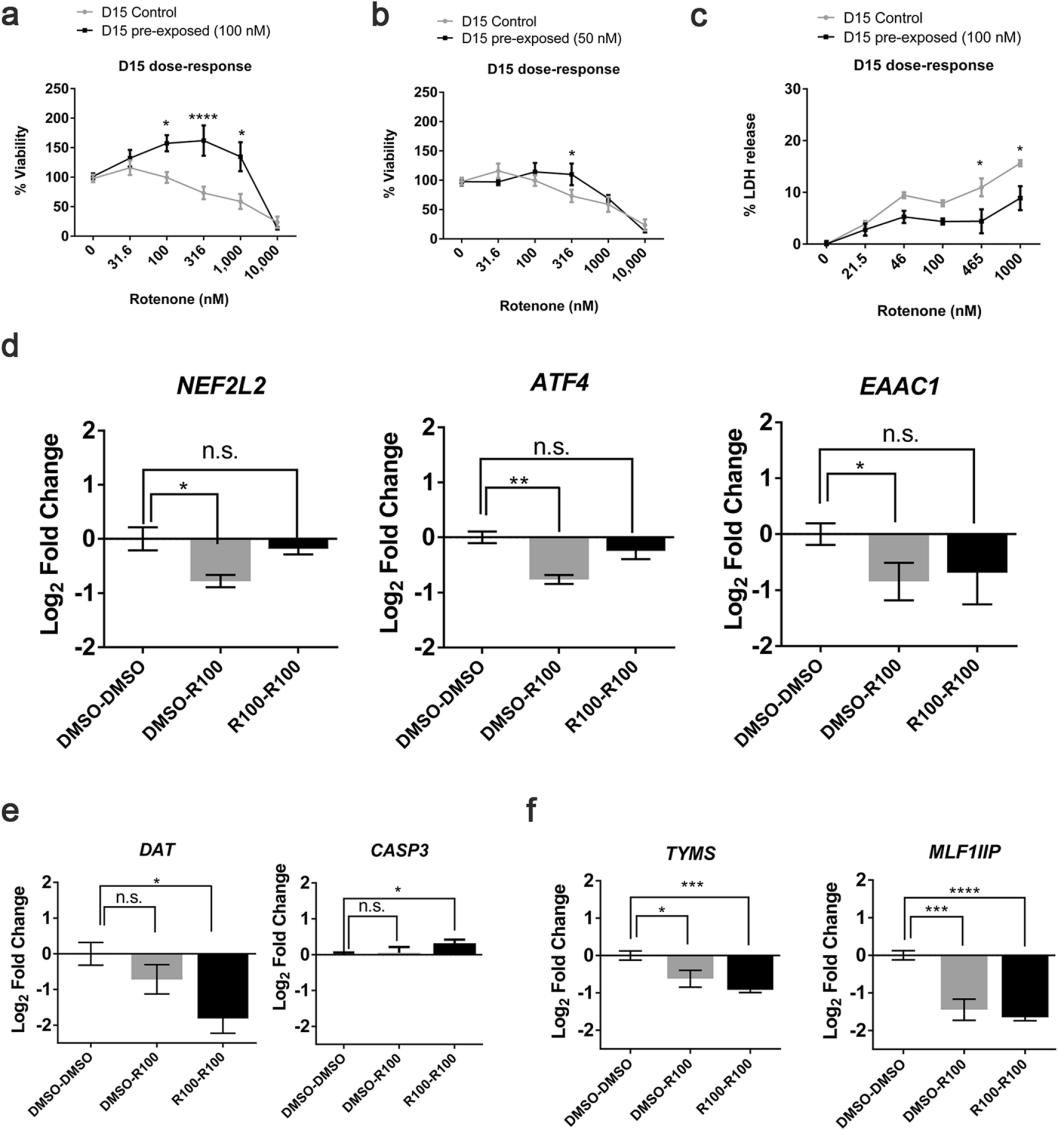
Effects of second exposure on viability and gene expression. **a, b** Cell viability concentration–response for aggregates on day 15, which were pre-exposed to DMSO (control) or rotenone (pre-exposed 100 or 50 nM) on day 8. **c** LDH-release dose–response for aggregates on day 15 that were pre-exposed to DMSO (control) or rotenone (pre-exposed 100 nM) on day 8. Dose–response curves were generated from three independent experiments and analyzed by one-way ANOVA followed by Bonferroni’s correction. **d** *NEF2L2, ATF4, EAAC1*, **e** *DAT, CASP3*, and **f** *TYMS, MLF1IP* gene expression measured by QT-PCR from three independent experiments and analyzed for significance using the Student’s *t* test and Bonferroni’s correction for multiple hypothesis testing. A *p* value < 0.05 is denoted by *, *p* < 0.01 by **, and *p* < 0.001 by ***, respectively

After observing that aggregates compensated for the inhibition of complex I and functionally recovered after the first insult, we tested our resilience hypothesis (Smirnova et al. 2015), by measuring susceptibility of pre-exposed 3D LUHMES to a second exposure to rotenone. After recovery from 100 nM rotenone treatment, aggregates were re-exposed on day 14 to increasing rotenone concentrations (0–10 µM). Viability was assessed 24 h later—on day 15. Control LUHMES aggregates, exposed to rotenone for the first time on day 14 (control), showed a similar dose–response (Fig. 8a) on day 15 to that previously observed on day 8 (published data Smirnova et al. 2016), indicating that the dopaminergic-cell response to rotenone in this model does not change between days 8 and 15 of differentiation. In contrast, aggregates pre-exposed to 100 nM rotenone on day 8 and re-exposed on day 14 (pre-exposed), showed a significant increase in viability/metabolic activity at concentrations between 100 nM and 1 µM (Fig. 8a) compared to controls. The IC_20_ increased from 260.36 to 5057.77 nM when cells were pre-exposed to 100 nM rotenone. To determine, whether observed effects were concentration-dependent, we pre-treated the aggregates on day 7 for 24 h with 25 and 50 nM rotenone. Aggregates pre-exposed to 50 nM also showed increased viability on day 15 compared to DMSO controls at 316 nM (Fig. 8b), while those pre-exposed to 25 nM were more similar to controls in response to the second hit (Supplemental Figure S5) (an IC_20_ of 259.62 and 812.95 nM were calculated for 25 and 50 nM pre-exposed cells). Thus, we concluded that the observed effect was also concentration-dependent as pre-exposure to 50 and 100 nM, but not 25 nM, led to increased viability (metabolic activity) and IC_20_ upon a second hit. Pre-exposed samples (100 nM) also showed lower level of released LDH than controls at higher concentrations (46 to 1000 nM) (Fig. 8c), confirming resilience of pre-exposed aggregates to the second hit.

### A second exposure elicits a different gene expression pattern

To further understand the changes taking place after a second exposure to rotenone, we measured changes in expression of genes previously shown to be altered by rotenone (Krug et al. 2014; Smirnova et al. 2016) as well as genes specific for dopaminergic neurons and PD. Gene expression was analyzed in three independent experiments for the following conditions: control cultures, never exposed to rotenone (DMSO–DMSO); cultures exposed to 100 nM rotenone on day 14 for the first time (DMSO-R100); cultures pre-exposed to 100 nM rotenone on day 7 and re-exposed on day 14 (R100-R100). Samples for qPCR were collected on day 15. *NEF2L2, ATF4*, and *EAAC1* were significantly downregulated when aggregates were exposed to rotenone for the first time on day 14 (Fig. 8d, DMSO–R100). However, no perturbation in expression of those genes was observed if aggregates were pre-exposed to rotenone on day 7 (Fig. 8d, R100–R100). On the contrarily, expression of DAT and CASP3 was not perturbed in aggregates exposed acutely to rotenone only on day 14 (Fig. 8e, DMSO–R100), but was significantly changed in pre-exposed samples (Fig. 8e, R100–R100). Similar downregulation of TYMS and MLF1IP (previously shown to be downregulated by both rotenone and MPP+) was observed in both conditions whether aggregates had been pre-exposed or not (Fig. 8f). These data showed that the genetic response varied depending on whether aggregates were exposed for the first time or second time.

## Discussion

Previously, we hypothesized that low-dose short-term stress can lead to several cellular outcomes (death, recovery, resilience, or increased susceptibility) (Smirnova et al. 2015). If cell death is not induced, cells that recover may have molecular signatures, which lead to resilience or susceptibility to a second hit. With the experiments described here, we have demonstrated that 3D LUHMES can restore functionality after the low-dose short-term (100 nM, 24 h) exposure to rotenone. First, we measured free rotenone concentration in medium prior to treatment, after 24 h treatment and 7 day post wash-out. These data showed that from 78.8 ng (100 nM), 23.5 ng bound to plastic, and 21.1 ng bound to cells. We further confirmed that by transferring aggregates to a new plate and performing a wash-out step on day 8, no further rotenone was present in the culture medium on day 15 (Fig. 1d, e). Although rotenone could remain bound to cells, and this would have to be measured to determine whether rotenone remains available to cells after wash-out, there was no further exposure in to rotenone from the medium during recovery.

We observed an acute and delayed decrease in cell viability after compound wash-out (D8 82.6%, D10 89.4%, D12 69.0%, D15 72.3%) (Fig. 2a). A 25% cell loss on day 15 was confirmed by DNA and protein quantification (Fig. 2c, d). The focus of this study was to determine what occurs to cells that survive the first exposure to rotenone. To assess whether surviving cells were metabolically affected after the cell viability stabilizes, we assessed complex I activity, ATP production, and mitochondria morphology after acute exposure and wash-out. Then, we assessed whether functionality of the neurons could be restored after compound wash-out by measuring outgrowth of neurites out from the aggregates. Delayed effects of rotenone on electrical activity were assessed after wash-out and recovery. Acute and delayed effects of rotenone on gene expression were analyzed by microarray. Finally, we measured the cellular response to a second-rotenone exposure after the recovery period.

To study recovery, we selected the LOAEL for rotenone as reported in 2D and 3D LUHMES cultures (Krug et al. 2014; Smirnova et al. 2016). Although it is suggested that culturing in 3D increase survival vs. 2D cultures (Alépée et al. 2014), culturing LUHMES in 3D did not increase cell survival to rotenone exposure, likely because rotenone is lipophilic and can easily diffuse to the center of aggregates. A lower concentration (50 nM) was found to alter gene expression but had no effect on other molecular and functional endpoints (data not shown), therefore, was only included in microarray analysis. Our previous work also found no difference in day 15 viability after 12 or 24 h exposure on day 7 and subsequent wash-out, but further toxicity after 48 h exposure and subsequent wash-out; therefore, 24 h was selected as the most relevant time-point for this study (Smirnova et al. 2016). Rotenone was shown to inhibit mitochondria complex I and studies have demonstrated that this inhibition is necessary for dopaminergic toxicity (Sherer et al. 2003); while others have shown off-target effects prior to complex I inhibition (Choi et al. 2008). Recovery and resilience have not yet been studied in a 3D in vitro human dopaminergic model, which is suitable for wash-out experiments (Smirnova et al. 2016; Alépée et al. 2014). Acutely, we observed a decrease in complex I activity (24 h) as observed in the previous studies (Choi et al. 2011; Richardson et al. 2005). Activity remained inhibited on day 15 (Fig. 3a), indicating that this effect was permanent after wash-out. We must note that this could be due to rotenone remaining bound within aggregates on day 15. An alternative explanation is damage to complex I or decreased expression of complex I subunits. Irreversible complex I inhibition has been shown previously (Lindahl and Öberg 1961). Acute reduction in ATP production has been well documented (Li et al. 2003; Sherer et al. 2003; Krug et al. 2014) and was confirmed in the present study, but in vitro models have not been able to demonstrate whether this is reversible. After rotenone wash-out, we observed an increase in ATP production on day 15, indicating that cells overcame complex I inhibition and have increased energy metabolism 7 days after compound removal (Fig. 3b). It is known that cells can shift from aerobic to anaerobic respiration to compensate for a decrease in ATP production in response to environmental stress (Zeiger et al. 2010). Zeiger et al. demonstrated that neurons will enhance ATP production following mild stress to survive. As multiple neuronal processes require ATP, an increase in ATP production may be necessary to recover cellular homeostasis in surviving cells. Dopaminergic neurons also have shown to have a large glycolytic spare capacity which could help to overcome lower ATP levels (Delp et al. 2017).

Upon studying mitochondrial morphology, our experiments showed a reversible increase in mitochondria diameter (Fig. 4). We have previously reported acute loss of mitochondrial membrane potential (quantified using MitoTracker^®^; Smirnova et al. 2016) which could lead to fission defects. Studies have shown that inhibition of mitochondrial fission or promotion of mitochondrial fusion has protective effects in rotenone-induced neurotoxicity (Peng et al. 2017), and studies have documented rotenone-induced effects on mitochondrial trafficking and movement (Fang et al. 2016; Haddad and Nakamura 2015; Borland et al. 2008). Mitochondria undergo dynamic changes using fusion and fission to maintain function and morphology during stress (Knott et al. 2008). Imbalances in these mechanisms and fragmented mitochondria have been found in PD patients and only recently in vitro (Reddy 2008; Peng et al. 2017). The analysis of mitochondria number also allowed us to confirm that the differences observed in complex I activity and ATP were not due to changes in the number of mitochondria.

Measuring neurite outgrowth is a common functional endpoint to test adverse effects of compounds on neuronal cells (Stiegler et al. 2011; Scholz et al. 2011; Sun et al. 2016). Neurite outgrowth requires ATP; therefore, the decrease in ATP production could be the reason for impaired outgrowth observed on day 8 (Fig. 5). In addition, the production of reactive oxygen species, which has previously been reported at this concentration, likely also plays a role (Li et al. 2003; Han et al. 2014). The inhibition of complex I leads to electron leaking and a higher number of free electrons are, therefore, available to react with molecular oxygen to produce O_2_^−^. It has also been shown that oxidative stress, induced by rotenone (Sherer et al. 2003), increases microtubule disruption (Ren et al. 2005; Feng 2006; Choi et al 2011). As observed with ATP production and mitochondria diameter, after the 7 day recovery period, neurite outgrowth was restored suggesting functional recovery, even while complex I remained inhibited.

LUHMES monolayer cultures have shown to be electrically active (Scholz et al. 2011), but this had not yet been studied in 3D LUHMES cultures. Patch clamp on day 15 revealed that 3D LUHMES aggregates are made up of both phasic and tonic (Fig. 6) dopaminergic neuronal cell types. It has been shown that dopaminergic neurons can be either of these types resulting in different amounts of dopamine release in the striatum (Vandecasteele et al. 2005). There were a higher number of phasic cells, which were further analyzed, and no difference in input resistance (*R*_m_) or minimal spike latency (Fig. 6d, e) was observed between treated and control samples on day 15. Together, these functional endpoints confirm that acute effects on metabolic activity, ATP production, mitochondria, and neurite outgrowth are reversible, and no delayed effects on electrical activity are observed after rotenone wash-out.

Functional endpoints were further confirmed with whole-genome microarray analysis (Fig. 7). As expected, the transcriptome was significantly perturbed (708 genes) on day 8, immediately after rotenone exposure, but after compound wash-out global gene expression was close to control (no significantly changed genes after FDR correction, 107 prior to FDR correction). Ten genes were altered on both day 8 and day 15, suggesting that the pathways that they are involved in are permanently perturbed. Most of those ten genes are enriched in the brain. At least two of the downregulated genes (*CD200* and *CCK*) are strongly associated with PD pathology with a significant literature support (Wang et al. 2007; Nilsson et al. 2009). *CD200* was shown to be downregulated in the *substantia nigra* of aging rats and blocking of the CD200 receptor significantly increased susceptibility of dopaminergic neurons to rotenone (Wang et al. 2011). *CD200* downregulation is associated with induced inflammation in PD, since this gene has anti-inflammatory and neuroprotective properties in dopaminergic neurons by inhibiting microglia activation and release of ATP and inflammatory factors (Ren et al. 2016). *CCK* is enriched in the brain (FANTOM5 atlas, http://fantom.gsc.riken.jp), which regulates release of dopamine that affect dopamine-related behavior. Its polymorphism is associated with PD symptoms (Lenka et al. 2016; Fujii et al. 1999). *ACTA1*—actin alfa 1 skeletal muscles—was strongly upregulated on day 8 and still elevated on day 15. Although highly enriched in muscle tissue, *ACTA1* is expressed in developing brain, especially in mesencephalon in various vertebrate species (Bertola et al. 2008). It is suggested to regulate axonal guidance, cellular motility, and cytoskeleton, and is a hub in the regulatory network of *LRRK2*, a high-risk PD gene (Dusonchet et al. 2014). Since we observed recovery in neurite outgrowth, overexpression of this gene may support this result. GDF15—growth differentiation factor 15—is a secreted ligand of the TGF-beta (transforming growth factor-beta) superfamily of proteins. It is involved in the stress response after cellular injury. Elevation of *GDF15* is associated with tissue hypoxia, inflammation, acute injury, and oxidative stress (Wiklund et al. 2010). It is precarious to over-interpret single gene changes, but the fact that the ultimately identified a few genes are consistently involved in PD and neuronal processes, stresses that there may be causal involvement.

From our results, we can conclude that although LUHMES 3D cultures were able to recover from acute rotenone exposure at molecular and functional levels, there was permanent complex I inhibition which cells need to adapt to. Several questions remain to be answered: what is threshold of complex I inhibition for which dopaminergic neurons, can compensate for? How long can cells overcompensate for the loss in aerobic respiration and maintain “normal” functionality? How detrimental could it be for the cells to maintain this response in the long term? How do cells react to repeated exposures?

Yet, it is not clear why dopaminergic neurons are more susceptible to toxicity by compounds such as rotenone than other cell types (Haddad and Nakamura 2015; Schildknecht et al. 2017). Some hypotheses refer to the low number of dopaminergic neurons in the brain (~ 500,000 in healthy subjects) (Pakkenberg et al. 1991), axonal length (Surmeier et al. 2010), increased ATP demand (Haddad and Nakamura 2015), and increased susceptibility to ROS and role of dopamine in ROS production (Gaki and Papavassiliou 2014). Based on our previous hypotheses (Smirnova et al. 2015), the next question that we posed was whether pre-exposed aggregates respond differently to a second exposure compared to controls, which have not previously been treated with rotenone. From this experiment, two outcomes were possible: (1) the cells could become robust/resilient or (2) more sensitive. To test our hypothesis, aggregates were washed and allowed to recover for 6 days, and then exposed a second time to increasing concentrations of rotenone for 24 h on day 14 (Fig. 8a). Viable mitochondria have a reducing environment due to NADPH or NADH being present (O’Brien et al. 2000). NADPH dehydrogenase or NADH dehydrogenase enzymes reduce resazurin into the fluorescent product resorufin (Riss et al. 2013). For this reason, this assay is used to measure mitochondrial metabolic activity/cell viability. Our results described in Fig. 8a showed that mitochondrial metabolic activity in the aggregates pre-exposed to rotenone at 50 or 100 nM was higher than controls showing resilience to a second exposure to rotenone. Pre-exposure to 25 nM, however, did not lead to resilience, which means that the response to a second exposure is likely dependent on the concentration of the first exposure. Sherer et al. (2003) reported that Ndufs4^−/−^ (complex I accessory subunit) primary cells had increased NADH content but were more susceptible to rotenone toxicity. It will have to be further determined, which molecular signatures or possible epigenetic mechanisms lead to resilience and whether this is a short-term or long-term phenomenon. Further research is also needed to better understand whether the altered metabolic response is an adaptive response, making the cells robust; or rather detrimental, leading to a disease pathway in a long-term perspective. This experimental approach could provide quantitative data for different key events in adverse outcome pathways (AOPs).

To identify changes in gene expression after a second exposure compared to alterations observed after a single exposure, we assessed genes which had previously found to be altered by rotenone in LUHMES. Three genes were less sensitive to rotenone exposure on day 14 after being pre-exposed to rotenone on day 7, suggesting their role in resilience. *NEF2L2*, the gene coding for Nrf2, a protein involved in the oxidative stress response (Shih et al. 2005); *ATF4*, previously found altered by rotenone and involved in cell stress and proteasome inhibition (Krug et al. 2014; Smirnova et al. 2016); and *EAAC1*, responsible for glutamate uptake and found to be downregulated in PD models (Kinoshita et al. 2014; Zhang et al. 2016a, b). This could indicate that pre-exposed cells do not activate these response mechanisms upon a second exposure, and may be more resilient to the activation of specific pathways. Conversely these could be protective pathways, which the cell cannot activate upon a second exposure (point of no return; Krug et al. 2014), or have reached a tipping point (Schildknecht et al. 2017; Jennings et al 2004; Koppelstaetter et al. 2004). Our results suggest new questions as to where the threshold of an effect lies (Bal-Price et al. 2015, 2017a, b; Terron et al. 2018).

Conversely, we found genes, which were altered to a greater extent upon a second exposure compared with a single exposure on day 14. This was observed for the dopamine transporter *DAT* and calcium-mediated apoptosis protein *CASP3* (Fig. 8e). Furthermore, *MLF1IP*, the gene coding for a centromere protein involved in mitotic progression and transcriptional regulation; and *TYMS*, an enzyme involved in the synthesis of thymidine nucleotides for DNA repair and mitochondrial thymidylate biosynthesis were downregulated to the same level in single-exposed and pre-exposed aggregates (Fig. 8f). We confirmed the downregulation of *MLF1IP* and *TYMS* by 100 nM rotenone that has previously been reported and was permanent after wash-out (Krug et al. 2014; Smirnova et al. 2016). For these genes, a second hit did not lead to further downregulation, indicating that there is likely a threshold for their permanent downregulation. *TYMS* downregulation has been found to increase oxidative stress production as well as activate protective pathways in multiple cancer lines (Ozer et al. 2015; Xu et al. 2017) but has not been extensively studied in neurons.

These results show that second exposures lead to activation of different expression patterns and, therefore, wash-out and repeated exposures could provide more insight for adverse outcome pathways (Leist et al. 2017) and potential therapeutic targets. Further experiments are needed to study whether, in the long term, these pathways are detrimental or cells continue to confer resilience (Karatsoreos and McEwen 2013; Delp et al. 2017). The present work does not inform us on the specificity of rotenone in inducing resilience, since no other cell types or chemicals were tested yet, but as, with an AOP approach, demonstrates how events which can lead to adversity and their reversibility can be addressed. Some have studied repeated-dose chronic effects in vitro (Borland et al. 2008; Shaikh and Nicholson 2009; Gourov and Currran 2014), but not with a focus on recovery, adaptation, and resilience in dopaminergic neurons. Gene–environment interactions play an important role in neurodegeneration, e.g., in PD, and an altered genetic/epigenetic response to toxicants is thought to primarily drive sporadic PD (Miranda-Morales et al. 2017). In the context of resilience, epigenetic mechanisms may play a more crucial role, supported by the abolished transcriptional changes after compound wash-out and fact that epigenetics lay in the interplay between genetic and environmental interactions. Post-translational regulation may also be important as many PD-related genes are tightly regulated via phosphorylation and ubiquitination (Oueslati 2016; Nakazawa et al. 2016; Xu et al. 2015; Wani et al. 2015).

Although studies have focused on neuroprotective mechanisms in animal and in vitro models via silencing of pathways involved in degeneration or overexpression of neuroprotective pathways (Yacoubian et al. 2010; Zharikov et al. 2015; Zhang et al. 2016a, b; Basil et al. 2017; Lee et al. 2017; to name a few), the reversibility of morphological and functional endpoints has not been shown in cultured cells. Understanding changes, which occur after compound removal is not only a new approach as to how in vitro toxicity testing should be addressed but also is crucial to understand long-term toxicity. In the field of neurodegenerative diseases, there is a need to better understand the interplay between degenerative, adaptive, and protective pathways to identify complex gene–environment interactions and therapeutic targets. 3D in vitro models, which allow for repeated exposures and recovery periods, will better help to understand how low-dose exposures may lead to long-term disease. Furthermore, more complex multicellular test systems would help to identify the role of support cells such as astrocytes and microglia in recovery as well as how the differentiation stage or ‘age’ affects dopaminergic toxicity (Pamies et al. 2017, 2018).

Taken together, this study shows that cells which seem to be functionally ‘recovered’ from a toxicant hit retain some form of memory and are not the same anymore. This is largely neglected in the many acute high dose in vitro experiments reported. Cellular resilience and/or the ‘molecular scar’ concept in neurotoxicology and neurodegeneration can be compared to our immune system response, which develops memory and prior stimulation can lead to a different response to subsequent stimuli (Henn et al. 2011). The imprint from earlier exposures, which can manifest as either a molecular scar (rendering cells more sensitive), or resilience (more tolerant) needs to be considered to understand real-life exposures and measure risk. The demonstration of resilience here as a type of chemical tolerance would suggest that we might be overestimating toxic effects from commonly performed acute toxicity studies. It will be most interesting to see whether these phenomena are toxicant-selective, i.e., whether tolerance is observed only for the same toxicant or a class of toxicants or whether the cells are more robust in general. The model system presented here will allow the characterization of such mechanisms in the future.

## Electronic supplementary material

Below is the link to the electronic supplementary material.

Supplementary material 1 (PDF 2742 KB)

Supplementary material 2 (XLSX 347 KB)
