# Supplementary material for: Toxicity, recovery, and resilience in a 3D dopaminergic neuronal in vitro model exposed to rotenone
Source: Arch Toxicol. 2018 Jun 28;92(8):2587–606. doi: 10.1007/s00204-018-2250-8 (PMC6063347; doi:10.1007/s00204-018-2250-8)
Supplement: Supplementary file 1 — Supplementary material 1 (PDF 2742 KB) [file 204_2018_2250_MOESM1_ESM.pdf]

Archives of Toxicology

Toxicity, recovery, and resilience in a 3D dopaminergic neuronal *in vitro* model exposed to rotenone

Georgina Harris[1], Melanie Eschment[2], Sebastian Perez Orozco[3], J. Michael McCaffery[3], Richard Maclennan[4], Daniel Severin[5], Marcel Leist[2], Andre Kleensang[1], David Pamies[1], Alexandra Maertens[1], Helena T Hogberg[1], Dana Freeman[6], Alfredo Kirkwood[5,7], Thomas Hartung[1,2] and Lena Smirnova[1*]

[1] Center for Alternatives to Animal Testing (CAAT), Johns Hopkins Bloomberg School of Public Health, Baltimore, MD, USA
[2] Center for Alternatives to Animal Testing (CAAT) Europe and Doerenkamp-Zbinden Chair of in-vitro Toxicology and Biomedicine/Alternatives to Animal Experimentation, Department of Biology, University of Konstanz, Konstanz, Germany

[3] The Integrated Imaging Center, Department of Biology, Engineering in Oncology Center and The Institute for NanoBioTechnology, Johns Hopkins University, Baltimore, MD, USA

[4] Cyprotex Discovery Ltd, Macclesfield, UK

[5] The Mind/Brain Institute, Johns Hopkins University, Baltimore, MD, USA

[6] Department of Environmental Health and Engineering, Johns Hopkins University, Baltimore, MD, USA

[7] Department of Neuroscience, Johns Hopkins University, Baltimore, MD, USA

* Corresponding author: lsmirno1@jhu.edu, +1 410 614 4990

Supplementary Methods

3D LUHMES Differentiation

Cell Culture and Differentiation: The cell culture protocol was followed as described by (Krug et al. 2014 and Scholz et al., 2011). Flasks were coated with a coating solution. Coating solution was incubated over night at 37°C. Before flasks were used for cell culture, they were washed twice with sterile distilled water. LUHMES were cultured in a 75 cm² pre-coated flask, containing 12 mL proliferation medium. The Flask was incubated at 37 °C, 5% C02 and 95% humidity. When cells reached 70-80% confluency after 2-3 days, they were passaged. Medium was aspirated and 1 mL TrypIE™ was added for 3 min at 37 °C. After detachment LUHMES were re-suspended in DMEM/F12 Medium (15 mL). After transferring the cell suspension to a falcon tube, they were centrifuged for 3 min at 1000 rpm. The trypsin containing medium was aspirated and the pellet was resuspended in 10 mL of proliferation medium. Cells were counted using a cell counter and trypan blue. $2x10^6$ cells were transferred into a 75 cm² flask, or $4x10^6$ in a 175 cm² flask. At day 0, 3D differentiation was initiated as described by Harris et al., 2017 and Smirnova et al., 2016. Cells were detached and counted as described above. $5.5x10^5$ – $6x10^5$ cells were seeded to each well of a 6-well plate. Each well contained 2 mL of differentiation medium. It was necessary to stay in the given range of cell density for differentiation to prevent increased proliferation of LUHMES during later steps of differentiation. The 6-well plates were kept on a shaker at 80 rpm, with 50 mm orbit diameter in an incubator at 37 °C, 10% CO_2 and 95% humidity. Due to increased cell—cell interaction in 3D aggregates compared to LUHMES cultured in monolayers, proliferation is partly increased (this observation has also been made in confluent 2D cultures). Therefore, after 3 days of differentiation Taxol (the anti-proliferative compound Paclitaxel) was added to the 6-well plates to prevent proliferation within aggregates (Smirnova et al. 2016). Anti-proliferation medium is composed of differentiation medium and 20 nM Taxol. To change medium/ add anti-proliferation medium, the plate was shaken in slow circle motions to allow aggregates accumulate in the middle. The plate was tilted, 800 µL of the medium was removed and 1 mL of anti-proliferation medium was added. The volume of removed medium varied slightly to account for evaporation. Finally the 6-well plates were placed again in the incubator at 37 °C, 10% CO_2 and 95% humidity. On day 5 of differentiation, Taxol was washed out. The maximum of volume (leaving ca. 200 µL) was removed from each well and 2 mL of fresh, pre-warmed wash medium was added to the wells. This step was repeated one more time adding differentiation medium. Medium was exchanged on days 8, 10 and 12 by removing 1 mL and adding of 1.2 mL differentiation medium. All media preparations can be found in *Harris et al. 2017*.

Treatment and wash-out

Treatment in 6-well plate (day 7 treatment): rotenone stock (100 mM in DMSO) was defrosted on the day of treatment (day 7). Prior to treatment, the 100 mM rotenone stock was diluted 1:10,000 in differentiation medium and vortexed for 15 seconds. DMSO (100 %) was prepared in the same way, diluted 1:10,000 in differentiation medium (0.01 % DMSO) and vortexed for 15 seconds. To treat 3D LUHMES cells, 20 µL of the 10 µM rotenone solution was added in the 6-well plate containing 2 mL medium per well, to reach the desired final concentration (100 nM). The same volume of the 0.01 % DMSO solution was added for controls (non-cytotoxic). 6-well plates were placed on the gyratory shaker in an incubator with same settings as previously described. LUHMES were incubated with the rotenone or DMSO for 24h.

Treatment in 24-well plate (day 15 dose-response): rotenone stock (100 mM in DMSO) was defrosted on the day of treatment (day 14). The stock was diluted to 100 µM (1:1000). Then, a 2x serial dilution of the rotenone stock was prepared (63.2 nM, 200 nM, 632 nM, 2µM, 20 µM) in differentiation medium. A DMSO control was prepared in the same way as the highest rotenone concentration (final 0.002 % DMSO). Differentiated aggregates were retrieved from the incubator, shaken in circle motions and collected into a 15 mL tube. After allowing the aggregates to sink to the bottom of the tube, medium was aspirated and aggregates were suspend in 6 mL fresh differentiation medium. 250 µL of the aggregate-suspension was added to each well of the 24-well plate. Then, 250 mL of the 2x serial dilution was added to wells in triplicates. The 24-well plate was placed in an incubator at 37 °C, 10% CO_2 and 95% humidity for 24 h. The final dose-response concentration was 0, 31.6 nM, 100 nM, 316 nM, 1 µM and 10 µM.

Compound wash-out: after 24h of exposure in 6-well plates (day 8), the compound was washed out. Plates were taken from the incubator and shaken in small circle motions to allow aggregates to collect in the center of the plate. The plate was tilted and almost all medium was aspirated (leaving 200 µL). 2 mL wash medium was added to each well. Then 2 mL differentiation medium was added to each well of a newly labelled 6-well plate (transfer to a new plate is required as rotenone can adhere to plastic and could slowly release into new media). Aggregates in wash medium were then transferred to the new 6-well plates containing 2mL differentiation medium using a 200 µL pipette (as little media as possible was transferred). These steps were performed quickly to avoid aggregate clumping during washing. New plates containing washed aggregates were then returned to the incubator on the gyratory shaker.

Viability

Resazurin Assay: viability was measured as described in Harris et al., 2017. Fluorescence emission at 580-610 nm was measured and %-cell viability was calculated by comparing relative fluorescence units (RFU) of treated vs. control samples after subtracting blank values.

LDH assay: LDH was measured in the media in control and treated samples following manufacturer's instructions (CytoTox 96® NonRadioactive Cytotoxicity Assay, Promega). Briefly, 1 well was lysed as a positive control and 50 uL medium from each was transferred into a 96-well plate. 50 uL substrate was added and the plate was incubated at RT for 30 min. Then, 50 uL stop solution was added to stop the reaction and the absorbance was measured at 490 nm in a spectrophotometer. After blank subtraction, cytotoxicity was calculated by the following formula:

Percent cytotoxicity = 100*(Experimental LDH (OD490) /Maximum LDH (OD490))

DNA extraction and quantification

Collected cells were resuspended in 1x Tris-EDTA pH 8.0 (Quality Biological), 0.3 % (v/v) SDS (10%, Quality Biological), and Proteinase K (1mg/ml, Invitrogen). Cells were lysed overnight, rotating at 65 °C. Lysed cells were treated with 10 µg of RNAse A (Thermo Scientific) for 30 min at 37 °C. Following digestion, cells were transferred to heavy phase lock gel (Quantabio) and an equal volume of Phenol: Chloroform: Isoamyl (24:25:1, Sigma) was added to the gel and mixed with inversion. Cells were centrifuged at 12,000 x g for 15 min and the aqueous phase separated. DNA was precipitated using Ethanol (100%, Pharamco-AAPER), 2 % (v/v) Sodium Acetate (3M, Mediatech), and 0.3 % (v/v) Linear Acrylamide (Ambion). DNA was pelleted by centrifugation at 4,000 x g for 15 min, washed with 70% Ethanol, and resuspended in 10 mM Tris-Cl pH 8.0 (Qiagen). Eluted DNA was incubated at 37 °C for at least one hour prior to quantification. DNA quantification was performed using the Qubit dsDNA Broad Range Assay Kit (Invitrogen) and Qubit 2.0 Fluorometer (Invitrogen) according to manufacturer's instructions.

RNA extraction, reverse transcription, and real-time PCR

Total RNA was extracted using either TRIzol® Reagent (Life Technologies) and RNA Clean & ConcentratorTM- Kit (Zymo Research) or Mirvana microRNA isolation kit (for microarray analysis) following the manufacturer's instructions. RNA integrity was measured using the Nanodrop 2000 (ThermoScientific) UV–Vis Spectrophotometer (260 nm). Equal amounts of purified RNA (500 ng) were reverse transcribed to cDNA using random hexamer primers (Promega) and M-MLV reverse

transcriptase Kit (Promega) following the manufacturer's instructions. A DNAse treatment step was included in cDNA synthesis to ensure the elimination of DNA traces. The cDNA was diluted 1:5, and qRT-PCR was performed. Expression of genes was analysed using TaqMan gene expression assay (Life Technologies) and TaqMan FAST advance Master Mix (Life Technologies) or by using Fast SYBR Green master mix (Life Technologies) and primers listed in Supplementary Table S1. 18S and GAPDH were used as housekeeping genes for TaqMan and SYBR Green PCRs, respectively. All RT-PCRs were performed in duplicates on Fast Applied Biosystems 7500 System (Life Technologies) with the following thermal cycling parameters: SYBR® Green RT-PCR (95 °C for 20 s, followed by 40 cycles of 3 s at 95 °C and 30 s 60 °C); a melting curve step was included in SYBR Green reactions (95 °C for 15 s, 60 °C for 1 min, 95 °C for 15 s, and 60 °C for 15 s); TaqMan gene expression assay (95 °C for 20 s, followed by 40 cycles of 3 s at 95 °C and 30 s 60 °C). $2^{-\Delta\Delta Ct}$ method was used to calculate the fold changes. Data collected from three independent experiments were calculated as average log2-fold change in independent biological replicates ± SEM. Differences in treated and control samples were analyzsed for statistical significance using an unpaired t-test and Bonferroni correction. A p value <0.05 is denoted in graph by *, $p < 0.01$ by **, and $p < 0.001$ by ***, respectively.

Complex I Activity Assay

Mitochondria Isolation was performed on ice using the reagent-based method (Mitochondria Isolation Kit for Tissue and Cultured Cells, BioVision); Complex I activity using mitochondrial Complex I Activity Colorimetric Assay Kit, BioVision following manufacturer's instructions. Briefly, cells were collected by pelleting 3 wells from a 6-well plate, centrifuging at 600 x g for 10 min at 4°C and discarding the supernatant. 500 µL of mitochondria isolation buffer (with protease inhibitor cocktail (Sigma-Aldrich)) was then added to pellet, vortexed and placed on ice for 2 min. Then, 5 µL of Reagent A was added, vortexed for 5 sec and incubated on ice for 5 min, vortexing every min for 5 sec. The dissociated cells were then centrifuged at 7000 x g for 10 min at 4°C. The supernatant was discarded and the pellet was washed with mitochondria isolation buffer. The supernatant was carefully removed and mitochondria were re-suspended in storage buffer. The protein concentration was measured using BCA protein assay (1:20 sample to working reagent ratio) using a Nanodrop2000 spectrophotometer. Sample concentration was adjusted to 2 µg/µL using storage buffer. Sample protein concentration > 0.5 µg/µL is needed for complex I activity measurements. Complex I activity was measured following manufacturer's instructions (mitochondrial Complex I Activity Colorimetric Assay Kit, BioVision). Briefly, the standard curve was measured by diluting the complex I dye stock solution in complex I assay buffer. Absorbance

at 600 nm was measured. The reaction mix was them prepared and added to each well as described by the manufacturer. 2 µL (4 µg) mitochondrial samples were added to wells containing "sample mix" and "sample + inhibitor mix". NADH 1x working solution was prepared and 30 µL were added to each well using a multichannel pipette to avoid any differences in starting time for the reactions in the wells. Immediately, the plate was read at 600 nm on kinetic mode at 30 second intervals for 5 min at RT. The kinetic data collected was then analyzed using the following equation.

Reduced Complex Dye = Total Complex Dye (9 nmol/well) - Oxidized complex I Dye (as read from standard curve)

Sample Complex I Activity = Δ[reduced complex Dye concentration] / (Δt x p) (mUnits / µg)

Δ[reduced complex Dye concentration] = change in reduced Complex I Due concentration during Δt

Δt = t2 – t1 (min)

p = mitochondrial protein (µg)

Cellular ATP assay

ATP quantification was performed on day 8 (after toxicant treatment) and day 15 (after wash-out) of differentiation. The bioluminescence ATP Assay Kit (Thermofisher, A22066) was used to determine the amount of intracellular ATP in aggregates according to manufacturer's instructions. The ATP assay gives total cellular ATP content and informs about cellular respiration. Firstly, one well of aggregates for each condition was collected into an Eppendorf tube and washed once with cold PBS. All steps were performed on ice. Aggregates were allowed to sink to the bottom of the tube and all PBS was removed. 50 µL of whole cell lysis buffer (0.3 g NaCl, 1 mL Tris (1M), 1 mL 10% NP-40, 0.2 mL EDTA (0.5M), 17.8 mL dH_2O) was added to each tube and aggregates were pipetted up and down. Tubes were placed on ice for 20-30 min to allow for complete lysis. Samples were mixed to ensure lysis and centrifuged at 10,000 g for 5 min. The assay buffer was prepared following the manufacturer's instructions. Assay buffer (100 µL) was added to each well of a 96-well plate and 10 µL of each sample was added as well as positive (1, 5 and 10 µM ATP) and negative (lysis buffer) controls, in triplicate. The plate was kept in the dark at room temperature for 15 min. Luminescence was read using GloMax® 96 Microplate Luminometer (Promega). To normalize ATP measurements to cell number, total protein concentration was

used. Therefore, Pierce BCA Protein Assay Kit was used, as described in manufacturer's instructions (https://tools.thermofisher.com/content/sfs/manuals/MAN0011430_Pierce_BCA_Protein_Asy_UG.pdf). BCA Protein working reagent (1:50) was prepared. 200 µL were transferred into a 96-well plate (samples and blank in duplicates). For each sample 10 µL of cell lysis was added in duplicate as well as blank wells containing whole cell lysis buffer. 10 µL bovine serum albumin (BSA) standard protein concentrations was added to wells containing 200 µL BCA working reagent. Concentrations for the standard curve were 0, 0.25, 0.5, 0.75, 1, 1.5, 1.75 and 2 mg/ml BSA. The plate was incubated for 30 min at 37 °C.

Absorbance was measured at 570 nm using a spectrophotometer. Average luminescence values ± SEM was calculated from at least four biological replicates and technical duplicates. Differences in treated and control samples were analyzed for statistical significance using unpaired Student's t-test. A p value < 0.05 is denoted on graphs by *, $p < 0.0001$ by ***, respectively.

Neurite outgrowth imaging and Sholl analysis

RFP-LUHMES (Schildknecht et al. 2013) were differentiated and treated as described previously. Aggregates were collected into an Eppendorf tube and washed once using wash medium on day 8 or day 15. Wash Medium was removed and replaced with differentiation medium. Differentiation medium was added to each well of Matrigel™ (BD Biosciences) pre-coated, flat-bottom, black 24 or 96-well plates (ThermoScientific). 5-10 aggregates were seeded into each well in triplicates ensuring they were well spread out within the well for better image quantification. Plates were incubated for 24 h at 37 °C, 5% CO_2. After 24 h, media was removed and aggregates were washed twice with warm PBS. Aggregates were then fixed with 4% PFA, 1:10,000 Hoechst 33342 (Invitrogen, Molecular Probes) for 1 hour and washed three times with PBS. Confocal images of 5 aggregates per sample in three independent experiments were obtained for neurite outgrowth analysis using Sholl Image J Software. Images were obtained using the LSM Zeiss Confocor2000. 16-bit Images were obtained with 20x objective for blue (Hoechst 42333) and red (RFP-LUHMES) channels. Images were opened using Image J (Fiji Image J Open Source Software), converted to B&W and the threshold adjusted to allow software to identify individual neurites (the same threshold was kept for all images in each individual experiment). A straight line was drawn from the center of the aggregate to the most distal point of outgrowth. Then neurites were counted in shells starting at the surface of the aggregate, allowing the software to determine the start and the end for multiple shells (radii). The software then automatically detected the number of neurite

intersections per radius (set at 10 pixel widths) and the number of intersections (Y) vs distance from aggregate (X) was recorded, plotted and analyzed.

Supplementary Table S1 Primer sequences used for SYBRGreen RT-PCR

GENE	Forward sequence, 5’-3’	Reverse sequence, 3’-5’
ATF4	GGCTGGCTGTGGATGGGTTG	CTCCTGGACTAGGGGGGCAA
GAPDH	CACCATCTTCCAGGAGCGAGATC	GCAGGAGGCATTGCTGATGATC
CASP3	TGGTTTTCGGTGGGTGTG	CCACTGAGTTTTCAGTGTTCTC
TYMS	CAGCTTCAGCGAGAACCCAG	ACCTCGGCATCCAGCCCAAC
MLF1IP	TTTGTAAGGCAGCCATCGCC	CTGTGGCTCTAACCGAAGCA

Supplemental Figures

a

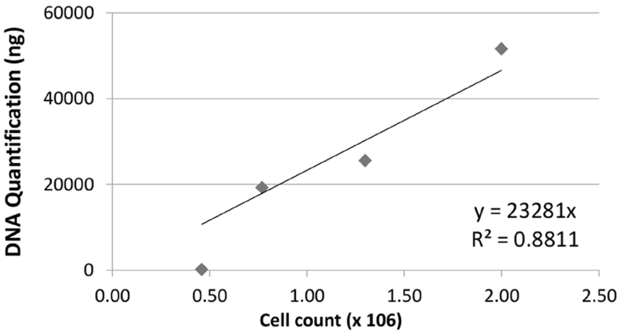

b

	D15 3D LUHMES		
	1 well	2 wells	3 wells
# aggregates	~200	~400	~800
DNA ng/ul	300	750	1140
Total DNA per sample (ng)	15000	37500	57000
DNA/aggregate (ng)	75	93.75	95
# cells/aggregate	3218.884	4023.605	4077.253

c

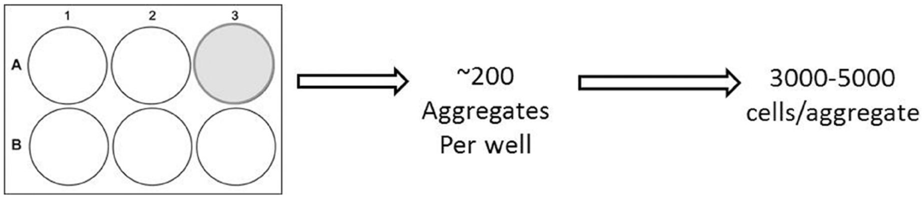

Suplimental Figure S1. DNA quantification and estimation of number of cells in one aggregate on day 15 of differentiation (Total DNA in sample ÷ 6 pg DNA per cell)

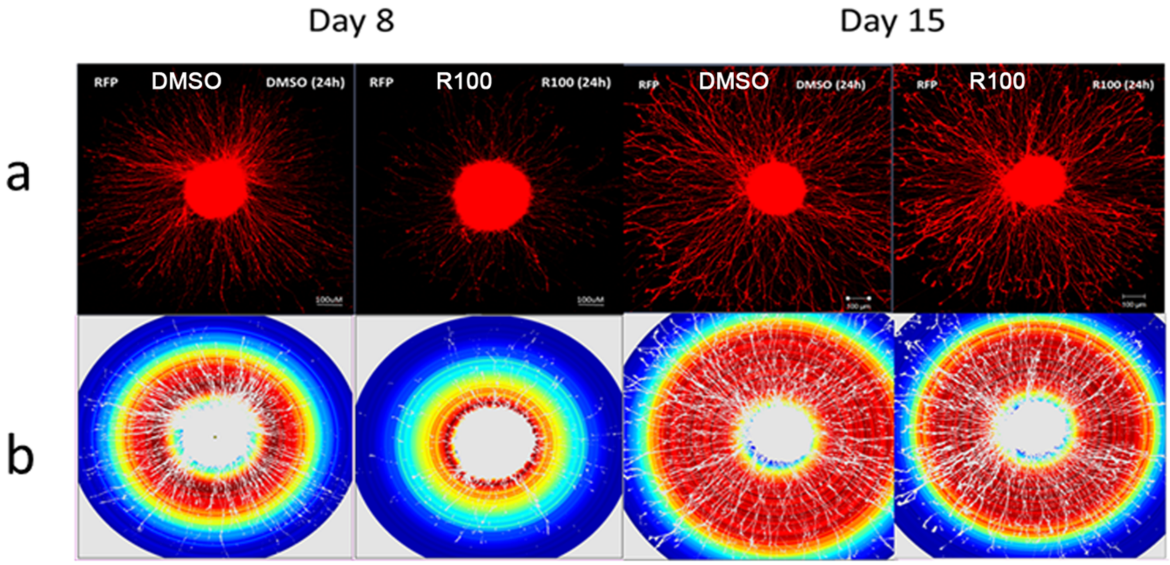

Supplemental Figure S2. Neurite Outgrowth analysis using Image J Sholl Analysis Software. (a) Sample images taken using a confocal microscope. (b) Analysis performed counting the number of intersections at each ring every 10 µm from the aggregate center. The number of intersections is marked from high (red) to low (blue). The data obtained gave number of intersections over distance. Red and blue indicate high and low number of intersections, respectively

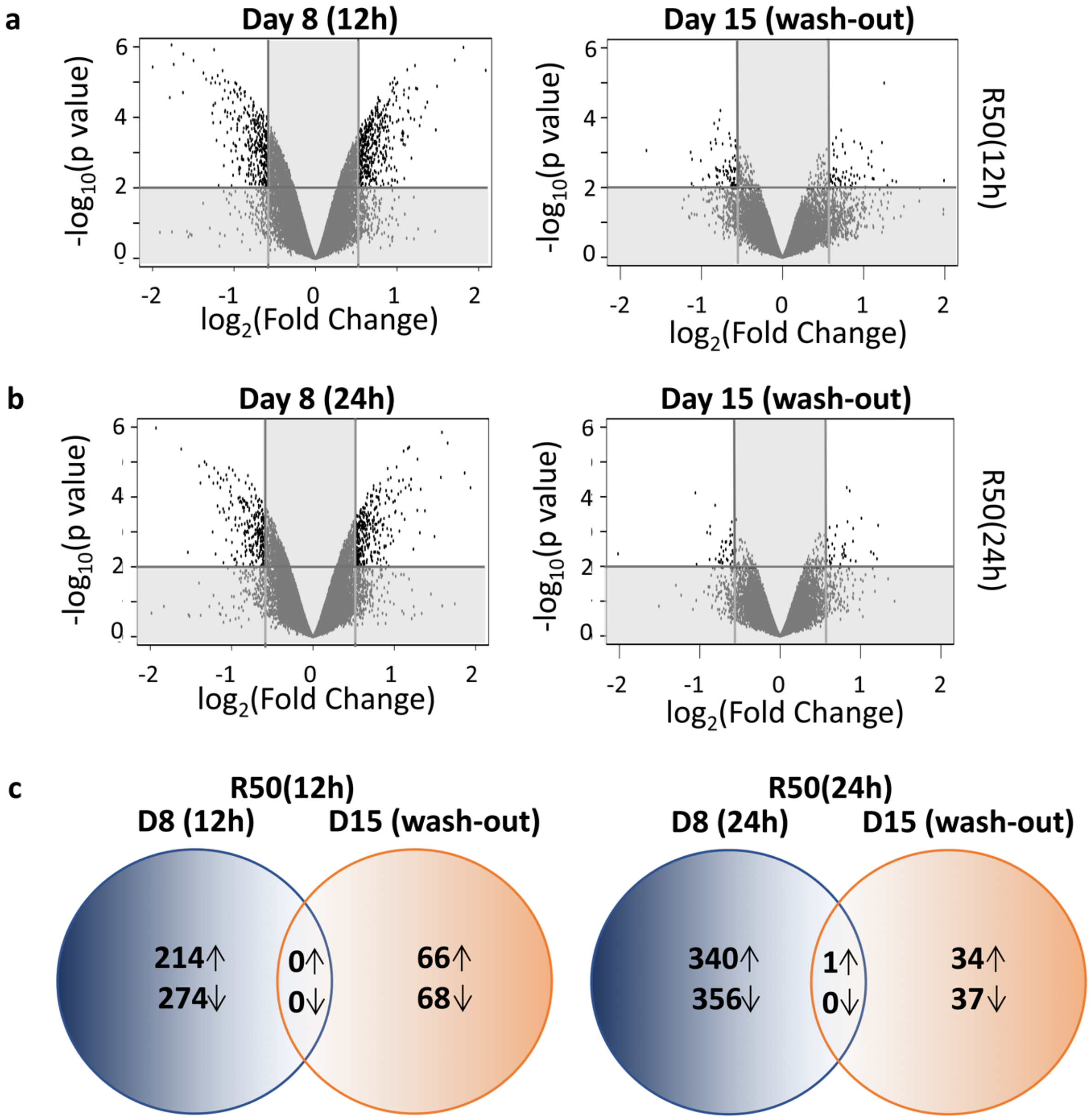

Supplemental Figure S3. 50 nM rotenone-induced transcriptome changes on day 8 (12 or 24h) vs. day 15 (wash-out). Volcano plots show significantly lower number of perturbed genes after compound wash-out and 7-day recovery period on day 15 vs. effects on day 8, after 12 h (a) or 24 h (b) exposure to 50 nM rotenone. (c) Venn-diagrams show the number of up- and down-regulated genes on day 8 [D8 (12 or 24h)] and on day 15 [D15 (wash-out)] (FC > 1.5, $p < 0.01$). One gene (FGFR3) was in intersection between two conditions in samples, treated with 50 nM for 24h

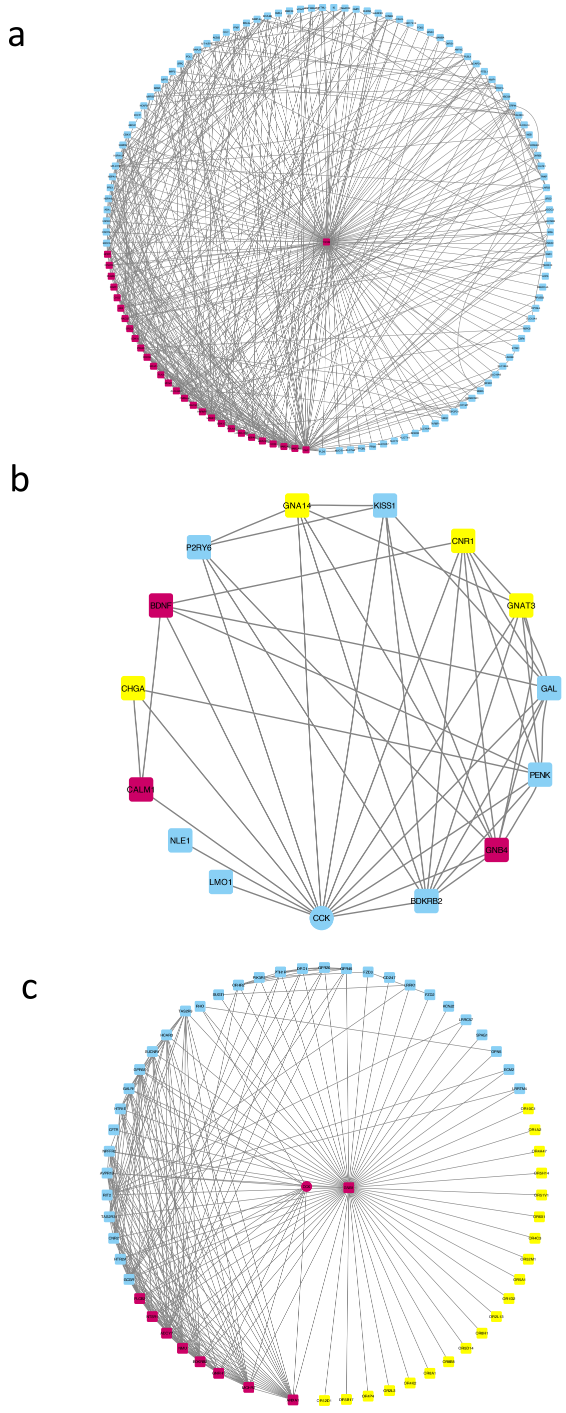

Supplemental Figure S4. All genes were visualized in the STRING interaction database for connections; all genes that are a "first neighbor" of (a) DNA topoisomerase II beta (TOP2B, the gene with the highest connection on day 8), (b) Cholecystokinin (CCK) on day 8 and (c) CCK and Guanine nucleotide-binding protein 1 (GNB1) subnetworks on day 15 are shown. Both subnetwork (b) and (c) were highly enriched for G-protein coupled receptor (GPCR, yellow). Genes with a degree higher than 20 (i.e. hubs) are indicated in magenta; genes in common between day 8 and day 15 are indicated with circles

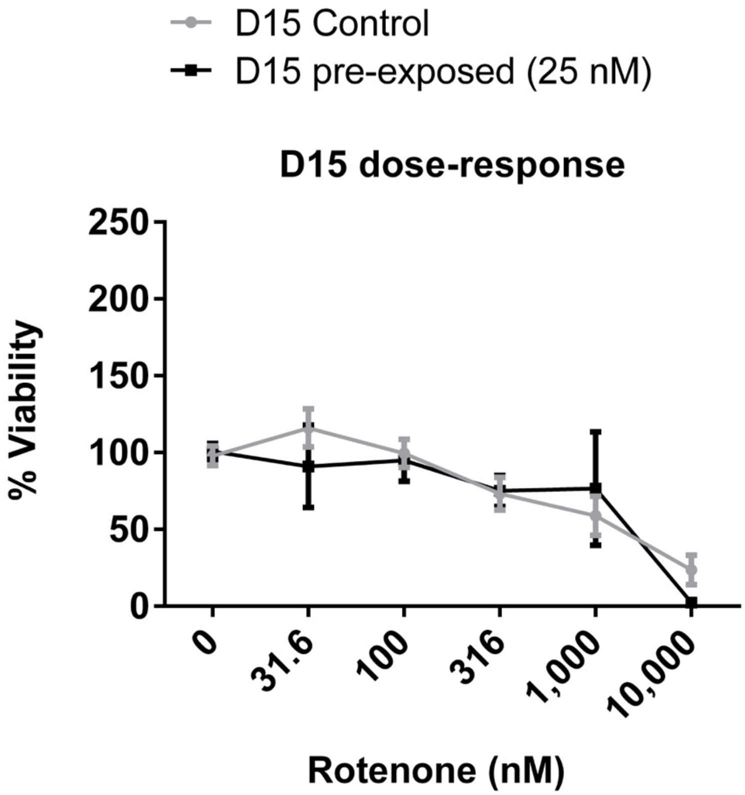

Supplemental Figure S5. Cell viability concentration-response for aggregates on day 15, pre-exposed to DMSO (Control) or rotenone (pre-exposed 25 nM) on day 8
